# Supplementary material for: Working lives of GPs in Scotland and England: cross-sectional analysis of national surveys
Source: BMJ Open. 2020 Oct 30;10(10):e042236. doi: 10.1136/bmjopen-2020-042236 (PMC7604859; doi:10.1136/bmjopen-2020-042236)
Supplement: Supplementary data [file bmjopen-2020-042236supp001.pdf]

SUPPLEMENTARY FILE

Table S1: Country differences in each domain of job satisfaction

Job satisfaction domain	Scotland (Mean, SD)	England (Mean, SD)	Unadjusted difference (Coeff, CI)	Adjusted difference (Coeff, CI)
Your colleagues and fellow workers	6.10 (1.14)	5.75 (1.23)	0.35 [0.26 to 0.45]	0.30 [0.20 to 0.39]
Amount of variety in your job	5.61 (1.20)	5.12 (1.38)	0.49 [0.39 to 0.60]	0.44 [0.34 to 0.55]
Amount of responsibility you are given	5.51 (1.43)	4.81 (1.68)	0.70 [0.57 to 0.83]	0.61 [0.48 to 0.74]
Physical working conditions	5.50 (1.45)	5.19 (1.45)	0.31 [0.19 to 0.42]	0.29 [0.18 to 0.41]
Opportunities to use your abilities	5.38 (1.26)	4.93 (1.45)	0.45 [0.34 to 0.56]	0.40 [0.29 to 0.52]
Freedom to choose your own method of working	5.34 (1.27)	4.78 (1.45)	0.56 [0.45 to 0.67]	0.53 [0.41 to 0.64]
Taking everything into consideration, how do you feel about your job	5.02 (1.34)	4.29 (1.49)	0.73 [0.62 to 0.85]	0.66 [0.54 to 0.78]
Your remuneration	4.93 (1.57)	4.28 (1.65)	0.65 [0.53 to 0.78]	0.60 [0.47 to 0.74]
Recognition you get for good work	4.72 (1.54)	4.41 (1.58)	0.31 [0.19 to 0.44]	0.28 [0.15 to 0.41]
Your hours of work	4.53 (1.66)	3.57 (1.79)	0.96 [0.82 to 1.10]	0.88 [0.74 to 1.03]

Notes: N=2,927. Satisfaction components are measured on a seven-point scale. Columns 4 and 5 are coefficients from linear regressions with robust standard errors. Adjusted differences control for age, gender, age x gender interaction, ethnicity, partner, and practice deprivation and urbanicity.

Table S2: Country differences in each job stressor

Job stressor	Scotland (Mean, SD)	England (Mean, SD)	Unadjusted difference (Coeff, CI)	Adjusted difference (Coeff, CI)
Increasing workloads	4.15 (0.93)	4.58 (0.68)	-0.42 [-0.48 to -0.36]	-0.41 [-0.47 to -0.35]
Increased demands from patients	4.01 (0.91)	4.29 (0.73)	-0.28 [-0.34 to -0.22]	-0.27 [-0.34 to -0.21]
Having insufficient time to do justice to the job	3.90 (1.04)	4.38 (0.83)	-0.48 [-0.55 to -0.41]	-0.47 [-0.54 to -0.39]
Dealing with problem patients	3.79 (0.97)	3.97 (0.88)	-0.17 [-0.24 to -0.10]	-0.16 [-0.24 to -0.084]
Paperwork	3.77 (0.97)	4.31 (0.87)	-0.54 [-0.62 to -0.47]	-0.52 [-0.59 to -0.44]
Finding a locum	3.61 (1.42)	2.98 (1.41)	0.63 [0.52 to 0.74]	0.75 [0.64 to 0.86]
Dealing with earlier discharges from hospital	3.58 (0.99)	3.88 (0.93)	-0.30 [-0.37 to -0.22]	-0.24 [-0.32 to -0.16]
Long working hours	3.47 (1.16)	4.10 (1.04)	-0.63 [-0.72 to -0.55]	-0.59 [-0.68 to -0.50]
Adverse publicity by the media	3.32 (1.25)	3.57 (1.22)	-0.25 [-0.34 to -0.15]	-0.21 [-0.31 to -0.11]
Unrealistically high expectation of role by others	3.30 (1.20)	3.75 (1.12)	-0.45 [-0.54 to -0.36]	-0.38 [-0.48 to -0.29]
Worrying about patient complaints/litigation	3.18 (1.13)	3.61 (1.12)	-0.44 [-0.52 to -0.35]	-0.38 [-0.48 to -0.29]
Insufficient resources within the practice	3.17 (1.16)	3.64 (1.15)	-0.47 [-0.56 to -0.38]	-0.38 [-0.47 to -0.28]
Interruptions by emergency calls during surgery	2.62 (1.10)	3.18 (1.11)	-0.57 [-0.66 to -0.48]	-0.52 [-0.61 to -0.43]

Notes: N=2,927. Stressors components are measured on a five-point scale. Columns 4 and 5 are coefficients from linear regressions with robust standard errors. Adjusted differences control for age, gender, age x gender interaction, ethnicity, partner, and practice deprivation and urbanicity.

Table S3: Country differences in each positive job attribute

Positive job attribute	Scotland (Mean, SD)	England (Mean, SD)	Unadjusted difference (Coeff, CI)	Adjusted difference (Coeff, CI)
My job provides me with a variety of interesting things	4.01 (0.73)	3.95 (0.78)	0.059 [-0.0019 to 0.12]	0.032 [-0.029 to 0.093]
I always know what my work responsibilities are	3.77 (0.85)	3.74 (0.93)	0.034 [-0.037 to 0.11]	0.0022 [-0.073 to 0.077]
I have a choice in deciding how I do my job	3.45 (0.94)	3.32 (1.00)	0.13 [0.051 to 0.21]	0.091 [0.011 to 0.17]
I am involved in deciding on the changes introduced that affect my work	3.17 (1.10)	3.24 (1.22)	-0.067 [-0.16 to 0.027]	-0.088 [-0.18 to 0.0088]
I have a choice in deciding what I do at work	3.10 (0.99)	2.97 (1.01)	0.13 [0.052 to 0.21]	0.10 [0.021 to 0.19]
I am consulted about changes that affect my work	3.08 (1.10)	3.01 (1.28)	0.072 [-0.025 to 0.17]	0.0052 [-0.096 to 0.11]
My working time can be flexible	2.81 (1.06)	2.92 (1.05)	-0.11 [-0.19 to -0.026]	-0.12 [-0.21 to -0.037]
I get clear feedback about how well I am doing my job	2.70 (0.92)	2.86 (0.96)	-0.16 [-0.23 to -0.082]	-0.13 [-0.21 to -0.049]
Changes to my job in the last year have led to better patient care	2.60 (0.95)	2.36 (0.97)	0.24 [0.17 to 0.32]	0.22 [0.14 to 0.30]

Notes: N=2,927. Positive job attributes components are measured on a five-point scale. Columns 4 and 5 are coefficients from linear regressions with robust standard errors. Adjusted differences control for age, gender, age x gender interaction, ethnicity, partner, and practice deprivation and urbanicity.

Table S4: Country differences in each negative job attribute

Negative job attributes domain	Scotland (Mean, SD)	England (Mean, SD)	Unadjusted difference (Coeff, CI)	Adjusted difference (Coeff, CI)
I have to work very intensively	4.34 (0.73)	4.60 (0.62)	-0.26 [-0.31 to -0.21]	-0.26 [-0.32 to -0.21]
I have to work very fast	4.09 (0.83)	4.35 (0.76)	-0.26 [-0.33 to -0.20]	-0.26 [-0.33 to -0.20]
I am required to do unimportant tasks which prevent me completing more important ones	3.90 (0.97)	4.09 (0.97)	-0.19 [-0.27 to -0.11]	-0.18 [-0.26 to -0.10]
I do not have time to carry out all my work	3.78 (1.02)	4.16 (0.98)	-0.38 [-0.46 to -0.30]	-0.36 [-0.44 to -0.28]

Notes: N=2,927. Negative job attributes components are measured on a five-point scale. Columns 4 and 5 are coefficients from linear regressions with robust standard errors. Adjusted differences control for age, gender, age x gender interaction, ethnicity, partner, and practice deprivation and urbanicity.

Table S5: Summary statistics for the covariates showing representativeness of the two surveys

	Scotland		England	
Variable names	Survey GPs (n=2,048) %	All GPs (Sep 2018) (n=4,366) %	Survey GPs (n=879) %	All GPs (Dec 2017) (n=34,549 %
Female	58%	58%	51%	54%
GP Partner	84%	78%	87%	66%
Black, Asian and Minority Ethnicity	9%	n/a	20%	n/a
Age category:				
<35 years	10%	13%	5%	12%
35-44 years	34%	34%	26%	34%
45-54 years	36%	33%	38%	31%
55-64 years	19%	18%	28%	19%
65+ years	1%	1%	3%	4%
Proportion of patients in an urban area	65%	66%	77%	81%
Proportion of patients in most deprived quintile	20%	19%	16%	19%

Notes: All data are presented for salaried and partnered GPs only. This is with the exception of population figures for deprivation and urbanicity indicators for Scotland, as GP headcount data by practice used to create this figure is only available for all GPs (including registrars, retainers and locums).

For deprivation, we use data on the number of patients living in each deprivation quintile registered with each practice. For Scotland, number of patients in each deprivation quintile is publicly available. For England, this is created using data on LSOA level quintiles of IMD and the number of patients in each LSOA.

For urbanicity, we use the distribution of patients in urban and rural areas. For Scotland, number of patients in each practice in each urban/rural classification is publicly available. For England this was created using the 2011 urban/rural classification by LSOA and the number of patients in each practice in each LSOA.

Ethnicity data is unavailable at the population level.

Table S6: Restricted sample analysis of differences between Scotland and England in intentions to reduce work participation

	Scotland (Mean, SD)	England (Mean, SD)	Unadjusted difference (Coef., 95% CI)	Adjusted difference (Coef., 95% CI)
Reduce your work hours within five years?	2.84 (1.58)	3.54 (1.49)	-0.70 [-0.84 to -0.55]	-0.53 [-0.66 to -0.39]
Leave medical work entirely within five years?	2.20 (1.53)	2.76 (1.63)	-0.55 [-0.70 to -0.41]	-0.31 [-0.42 to -0.20]
Leave direct patient care within five years?	2.33 (1.54)	2.93 (1.59)	-0.60 [-0.74 to -0.45]	-0.36 [-0.47 to -0.24]
Continue with medical work but outside the UK within five years?	1.37 (0.79)	1.61 (1.03)	-0.25 [-0.33 to -0.16]	-0.22 [-0.30 to -0.13]

Notes: N=1,800. Intentions to reduce work participation indicators are measured on a five-point scale. Columns 4 and 5 are coefficients from linear regressions with robust standard errors. Adjusted differences control for age, gender, age x gender interaction, ethnicity, partner, and practice deprivation and urbanicity.
Analysis is based on the first 25.2% of respondents in the Scottish sample to match the returned English sample.

Table S7: Restricted sample analysis of differences between Scotland and England across all satisfaction, job stressors, and job attributes domains

	Scotland (Mean, SD)	England (Mean, SD)	Unadjusted difference (Coef., 95% CI)	Adjusted difference (Coef., 95% CI)
Satisfaction	5.29 (0.98)	4.71 (1.07)	0.58 [0.49 to 0.68]	0.51 [0.41 to 0.61]
Job stressors	3.53 (0.78)	3.86 (0.66)	-0.33 [-0.40 to -0.26]	-0.28 [-0.35 to -0.21]
Positive job attributes	3.19 (0.60)	3.15 (0.62)	0.037 [-0.020 to 0.093]	0.0054 [-0.053 to 0.064]
Negative job attributes	4.01 (0.66)	4.30 (0.57)	-0.29 [-0.35 to -0.23]	-0.28 [-0.34 to -0.22]

Notes: N=1,800. Satisfaction is measured 1-7. Job stressors and job attributes are measured 1-5. Columns 4 and 5 are coefficients from linear regressions with robust standard errors. Adjusted differences control for age, gender, age x gender interaction, ethnicity, partner, and practice deprivation and urbanicity.
Analysis is based on the first 25.2% of respondents in the Scottish sample to match the returned English sample.
