# Supplementary material for: Working lives of GPs in Scotland and England: cross-sectional analysis of national surveys
Source: BMJ Open. 2020 Oct 30;10(10):e042236. doi: 10.1136/bmjopen-2020-042236 (PMC7604859; doi:10.1136/bmjopen-2020-042236)
Supplement: Author's manuscript [file bmjopen-2020-042236.draft_revisions.pdf]

BMJ Open

BMJ Open is committed to open peer review. As part of this commitment we make the peer review history of every article we publish publicly available.

When an article is published we post the peer reviewers' comments and the authors' responses online. We also post the versions of the paper that were used during peer review. These are the versions that the peer review comments apply to.

The versions of the paper that follow are the versions that were submitted during the peer review process. They are not the versions of record or the final published versions. They should not be cited or distributed as the published version of this manuscript.

BMJ Open is an open access journal and the full, final, typeset and author-corrected version of record of the manuscript is available on our site with no access controls, subscription charges or pay-per-view fees (http://bmjopen.bmj.com).

If you have any questions on BMJ Open's open peer review process please email info.bmjopen@bmj.com

Working lives of GPs in Scotland and England: cross-sectional analysis of national surveys

Journal:	*BMJ Open*
Manuscript ID	bmjopen-2020-042236
Article Type:	Original research
Date Submitted by the Author:	30-Jun-2020
Complete List of Authors:	Hayes, Helen; The University of Manchester<br>Gibson, Jonathan; University of Manchester,<br>Fitzpatrick, Bridie; University of Glasgow, Institute of Health and Wellbeing<br>Checkland, Kath; The University of Manchester, Institute of Population Health; Centre for Biostatistics<br>Guthrie, Bruce; University of Edinburgh College of Medicine and Veterinary Medicine,<br>Gillies, John; The University of Edinburgh, The Usher Institute of Population Health Sciences and Informatics<br>Sutton, Matt; The University of Manchester<br>Mercer, Stewart; The University of Edinburgh, The Usher Institute of Population Health Sciences and Informatics
Keywords:	PRIMARY CARE, Health policy < HEALTH SERVICES ADMINISTRATION & MANAGEMENT, Organisation of health services < HEALTH SERVICES ADMINISTRATION & MANAGEMENT

I, the Submitting Author has the right to grant and does grant on behalf of all authors of the Work (as defined in the below author licence), an exclusive licence and/or a non-exclusive licence for contributions from authors who are: i) UK Crown employees; ii) where BMJ has agreed a CC-BY licence shall apply, and/or iii) in accordance with the terms applicable for US Federal Government officers or employees acting as part of their official duties; on a worldwide, perpetual, irrevocable, royalty-free basis to BMJ Publishing Group Ltd ("BMJ") its licensees and where the relevant Journal is co-owned by BMJ to the co-owners of the Journal, to publish the Work in this journal and any other BMJ products and to exploit all rights, as set out in our licence.

The Submitting Author accepts and understands that any supply made under these terms is made by BMJ to the Submitting Author unless you are acting as an employee on behalf of your employer or a postgraduate student of an affiliated institution which is paying any applicable article publishing charge ("APC") for Open Access articles. Where the Submitting Author wishes to make the Work available on an Open Access basis (and intends to pay the relevant APC), the terms of reuse of such Open Access shall be governed by a Creative Commons licence – details of these licences and which Creative Commons licence will apply to this Work are set out in our licence referred to above.

Other than as permitted in any relevant BMJ Author's Self Archiving Policies, I confirm this Work has not been accepted for publication elsewhere, is not being considered for publication elsewhere and does not duplicate material already published. I confirm all authors consent to publication of this Work and authorise the granting of this licence.

For peer review only

Working lives of GPs in Scotland and England: cross-sectional analysis of national surveys

Ms Helen Hayes MA, MSc

Research Assistant

Division of Population Health, Health Services Research & Primary Care, University of Manchester, Oxford Road, Manchester M13 9PL

Dr Jonathon Gibson BA, PG Dip, MSc, PhD

Research Fellow

Division of Population Health, Health Services Research & Primary Care, University of Manchester, Oxford Road, Manchester M13 9PL

Dr Bridie Fitzpatrick MSc, PhD

Honorary Senior Research Fellow, Institute for Health and Wellbeing, University of Glasgow, Glasgow G12 9LX

Professor Katherine Checkland BMedSci, MBBS, MA, PhD, MRCGP

Professor of Health Policy and Primary Care, Division of Population Health, Health Services Research & Primary Care, University of Manchester, Oxford Road, Manchester M13 9PL

Professor Bruce Guthrie BA, MB BChir, MSc, PhD, MRCP, FRCGP, FRSE

Professor of General Practice, Centre for Populations Health Sciences, Usher Institute, University of Edinburgh, Teviot Place, Edinburgh EH8 9AG

Professor Mathew Sutton BA, MSc, PhD

Chair in Health Economics, Director of Applied Health Research Domain, Division of Population Health, Health Services Research & Primary Care, University of Manchester, Oxford Road, Manchester M13 9PL

Professor John Gillies OBE MBChB, MA, FRSE, FRCGP, FRCPE

Deputy Director of the Scottish School of Primary Care, Honorary Professor in General Practice, Centre for Populations Health Sciences, Usher Institute, University of Edinburgh, Teviot Place, Edinburgh EH8 9AG

Professor Stewart W Mercer BSc (Hons), MBChB, MSc, PhD, FRCGP, FRCPE, FFPHM*

Director of the Scottish School of Primary Care, Professor of Primary Care and Multimorbidity, Centre for Populations Health Sciences, Usher Institute, University of Edinburgh, Teviot Place, Edinburgh EH8 9AG

*Corresponding author: stewart.mercer@ed.ac.uk

For peer review only

For peer review only - http://bmjopen.bmj.com/site/about/guidelines.xhtml

Abstract

Objectives

The UK faces major problems retaining general practitioners (GPs). Scotland introduced a new GP contract in April 2018, intended to better support GPs. This study compares the career intentions and working lives of GPs in Scotland with GPs in England, shortly after the new Scotland contract was introduced.

Design and setting

Comparison of cross-sectional analysis of survey responses of GPs in England and Scotland in 2017 and 2018 respectively, using linear regression to adjust the differences for gender, age, ethnicity, urbanicity and deprivation.

Participants

2,048 GPs in Scotland and 879 GPs in England.

Main outcome measures

Four intentions to reduce work participation (five-point scales: 1='none', 5='high'): reducing working hours; leaving medical work entirely; leaving direct patient care; or continuing medical work but outside the UK. Four domains of working life: job satisfaction (seven-point scale: 1='extremely dissatisfied', 7='extremely satisfied'); job stressors (five point-scale: 1='no pressure', 5='high pressure); positive and negative job attributes (five-point scales: 1='strongly disagree', 5='strongly agree').

Results

Compared to England, GPs in Scotland had lower intentions to reduce work participation, including a lower likelihood of reducing work hours (2.78 versus 3.54; adjusted difference = -0.52; 95%CI -0.64 to -0.41), a lower likelihood of leaving medical work entirely (2.11 versus 2.76;

For peer review only - http://bmjopen.bmj.com/site/about/guidelines.xhtml

adjusted difference = -0.32; 95%CI -0.42 to -0.22), a lower likelihood of leaving direct patient care (2.23 versus 2.93; adjusted difference = -0.37; 95%CI -0.47 to -0.27), and a lower likelihood of continuing medical work but outside of the UK (1.41 versus 1.61; adjusted difference = -0.2; 95%CI -0.28 to -0.12). GPs in Scotland reported higher job satisfaction, lower job stressors, similar positive job attributes, and lower negative job attributes.

Conclusions

Following the introduction of the new contract in Scotland, GPs in Scotland reported significantly better working lives and lower intention to reduce work participation than England.

For peer review only

Strengths and Limitations

- This is the first study to compare the working lives of GPs in Scotland and England using a set of common, validated questions on satisfaction, job characteristics and job pressures and using a similar data collection method.
- In both countries, largely nationally-representative samples were collected.
- We have adjusted the differences for respondents' age, gender, ethnicity and partner status, as well as the deprivation and urbanicity of the populations registered with the practices at which these GPs work.
- The response rate was lower to the England survey. Evidence suggests that less satisfied GPs are more likely to respond to job satisfaction surveys 24. If also true in this context, then the lower response rate in the English survey may mean it represents a pool of less satisfied English GPs and the differences could overestimate the true population differences.
- However, our supplementary analyses restricting the Scottish sample to the first 25.2% of returned surveys to match the total response rate in the English survey, showed only trivial differences compared to the full sample

For peer review only

Introduction

Low physician job satisfaction, stress and burnout are associated with increased staff turnover and threats to patient safety[1–4] and contribute to recruitment and retention problems in general practice in the United Kingdom (UK). General Practitioners' (GPs) job satisfaction fell markedly in the UK between 2012 and 2015[5] and in a recent survey of 11 countries, UK GP job satisfaction was second lowest.[6] The general practice 'crisis'[7] in the UK reflects a substantial increase in workload[8] and surveys of English GPs' working lives have shown an increasing proportion intending to leave direct patient care within 5 years, up to a high of 39% in 2017.[9]

Despite policy ambitions to increase the number of GPs in the UK, there was a 1.4% decrease in full-time equivalent GPs in England between September 2017 and September 2018.[10] Failure to address GP recruitment and retention puts a number of health policy goals at risk, including shifting care out of hospital and into the community.

Whereas England has emphasised market mechanisms and inspection regimes to assure quality, Scotland has placed the emphasis on collaboration and integration. England retains the Quality and Outcomes Framework (QOF) and has implemented Care Quality Commission inspections; Scotland abolished the QOF in 2016 and established GP Clusters, [11] as the forerunner to a radical new GP contract in April 2018.[12]

GP job satisfaction is an important indicator of the impact of GP contractual reform. We compare this between GPs in England in 2017 and Scotland in 2018 using cross-sectional survey data.

For peer review only - http://bmjopen.bmj.com/site/about/guidelines.xhtml

Methods

Design and setting

Data for England was from the 2017 National GP Worklife Survey,[9]using validated measures of satisfaction,[13]job pressure and attributes.[14] Data for Scotland was from the Scottish School of Primary Care survey and included the same validated measures.[15]

Ethical approval was granted for the English survey from the University of Manchester's Research and Ethics committee on the 5/10/2017 (Ref: 2017-2638-3884). The Scottish survey was assessed as not requiring ethical approval by the NHS or the University of Glasgow MVLS College Ethics Committee.

Participants

The English survey questionnaire was first posted to a random sample of 4,000 GPs in October 2017 with an option to complete an online version. Two reminders letters were sent in November and December 2017. Responses were returned by 996 GPs, representing 25.2% of the 3,953 eligible respondents after excluding GPs who had died, retired, left the practice or returned blank questionnaires.[9]

The Scottish survey questionnaire was first mailed to all 4,371 GPs in July 2018 which included the option of completing an online version, followed by two reminder paper mailings with further copies of the questionnaire in August and September 2018. Additionally, two further email reminders were sent (with the link to the online version) were sent mid and late August. The Scotland sample includes responses from 2,465 GPs (56.4% response rate). Due to the higher response rate the response rate, we ran supplementary analyses which restricted the sample in Scotland to the first 25.2% of returned surveys, to match the response rate in England.

The final sample consisted of 2,927 GPs (2,048 GPs in Scotland and 879 GPs in England) after removal of individuals with missing information on the outcome variables and covariates.

For peer review only - http://bmjopen.bmj.com/site/about/guidelines.xhtml

Outcome variables

We measured GPs' intentions to reduce work participation and four related domains of working lives; job satisfaction, job stressors, positive job attributes, and negative job attributes. For each domain, we created an average score for all of the questions for each respondent. Individual components are shown in the Supplementary file (Tables S1-S4).

Intentions to reduce work participation

We measured GPs intentions (in the next 5 years) to reduce work hours, leave medical work entirely, leave direct patient care, or to continue medical work but outside the UK, answered on on a 5-point scale with 1 indicating "none", 2 "slight", 3 "moderate", 4 "considerable" and 5 "high". We consider each of these components as separate outcomes.

Domains of working lives

The four domains of working lives were defined as follows:

Job satisfaction. Satisfaction with 10 different aspects of their job.[13] Ratings on a 7-point scale from 1 "extremely dissatisfied" and to 7 "extremely satisfied". The 10 answers were then averaged (minimum possible score of 1 and maximum possible score of 7).

Job stressors. Pressure experienceed from 13 job factors, rated on a 5-point scale; 1 "no pressure", 2 "slight pressure", 3 "moderate pressure", 4 "considerable pressure", 5 "high pressure". The 13 answers were averaged (minimum possible score of 1 and maximum possible score of 5).

Positive job attributes. Nine statements realting to 'positive' or desirable job aspects, where 1 indicates "strongly disagree", 2 "disagree", 3 "neutral", 4 "agree" and 5 "strongly agree". The nine answers were averaged (minimum possible score of 1 and maximum possible score of 5).

Negative job attributes. Four statements which relate to 'negative' or undesirable job aspects where 1 indicates "strongly disagree", 2 "disagree", 3 "neutral", 4 "agree" and 5 "strongly agree". The four answers were averaged (minimum possible score of 1 and maximum possible score of 5).

For peer review only - http://bmjopen.bmj.com/site/about/guidelines.xhtml

For peer review only

Covariates

We adjusted for: combinations of gender and age (five categories of 25-34, 35-44, 45-54, 55-64 and 65+ years); ethnicity (Black, Asian or minority ethnic group versus White ethnic group); practice urbanicity (proportion of patients living in an urban area,[16]and practice deprivation (proportion of patients living in an area in the most deprived quintile using an adjusted Index of Multiple Deprivation for England to ensure comparability with Scotland.[17]

Summary statistics and representativeness

The age, gender and partner status of respondents were compared to census data for GPs in England in December 2017[18] and in Scotland from the Information Services Division (ISD) Scotland in September 2018.[19] We also compared, separately for each country, the average proportions of patients in the most deprived quintile and in an urban area in the practices of the respondent GPs and all GPs. [16,19–22] The Scotland sample was more representative than the England sample, with women, younger doctors and non-partners being less under-represented in Scotland. GPs from more deprived and more urban practices were under-represented in the England sample (Supplementary file, Table S5). We adjust for these factors in the country comparisons.

Statistical analysis

Linear regression models with heteroscedasticity-robust standard errors to calculate point estimates and 95% confidence intervals for the differences of Scotland from England. In the adjusted models, age, gender, age and gender interaction, ethnicity, practice urbanicity and practice deprivation were included as covariates. We used average scores for the domains: job satisfaction, job stressors, positive job attributes and negative job attributes. The analysis was repeated on each separate question within these domains (see Supplementary file, Tables S1-S4). The questions were measured on ordered categorical scales and so we also checked the robustness of the adjusted

For peer review only - http://bmjopen.bmj.com/site/about/guidelines.xhtml

differences to the use of ordered probit regression models and results were qualitatively similar.

Results

Intentions to reduce work participation

GPs in Scotland reported lower likelihood of; reducing work hours (2.78 versus 3.54; adjusted difference -0.52; 95%CI -0.64 to -0.41), continuing medical work outside of the UK (1.41 versus 1.61; adjusted difference -0.2; 95%CI -0.28 to -0.12), leaving direct patient care (2.23 versus 2.93; adjusted difference -0.37; 95%CI -0.47 to -0.27), and leaving medical work entirely (2.11 versus 2.76; adjusted difference -0.32; 95%CI -0.42 to -0.22) compared with England (Table 1, Figure 1).

Domain scores

GPs in Scotland reported statistically significantly higher levels of satisfaction (5.27 versus 4.71; adjusted difference 0.50; 95%CI 0.42 to 0.59, lower levels of pressure (3.53 versus 3.86; adjusted difference -0.29; 95%CI -0.35 to -0.23), similar positive job attributes (3.19 versus 3.15; adjusted difference 0.01; 95%CI -0.04 to 0.06),) and less agreement in response to negative job attributes (4.03 versus 4.30; adjusted difference -0.27; 95%CI -0.31 to -0.22) (Table 2,

).

Scottish GPs had higher job satisfaction across all ten satisfaction domains, lower job stress for 12 of the 13 stressors (the exception being finding a locum), higher scores on six of the nine positive

For peer review only - http://bmjopen.bmj.com/site/about/guidelines.xhtml

job attributes (the exceptions being involvement in changes affecting their work, flexibility in working time and clear feedback about performance), and lower scores on all four of the negative job attributes (Figure 2). These differences remained in the adjusted regression analysis (see Supplementary file Tables S1-S4)

In both countries, almost every job stressor was (on average) rated in the range moderate to considerable. GPs in both countries disagreed or were neutral (on average) in their rating of five positive job attribute statements, with only slight average agreement in relation to having an interesting variety to work, knowing their responsibilities, having a choice in how to do the job, and being involved in changes introduced. On average, GPs in both countries agreed with all four negative job attribute statements (Figure 2) (see Supplementary file Tables S1-S4 for exact average values).

All differences between England and Scotland remained robust after controlling for GP characteristics, and practice urbanicity and deprivation. In the supplementary analyses, which restricted the sample in Scotland to the first 25.2% of returned surveys to match the total response rate in the English survey, there were only trivial differences in the results compared with those using the full sample (see Supplementary file Tables S6 and S7).

For peer review only - http://bmjopen.bmj.com/site/about/guidelines.xhtml

Discussion

Summary

This study has demonstrated that compared with GPs in England, GPs in Scotland have lower intentions to reduce work participation, as well as higher levels of job satisfaction, lower job stressors, and lower negative job attributes. These differences were of a reasonably large magnitude (one third to one half of a standard deviation), and thus likely to be meaningful in practice. It seems likely that these differences relate, at least in part, to the recent changes in primary care in Scotland, including the new GP contract and the abolition of the increasingly unpopular QOF [23].

However, notwithstanding these differences, GPs in both countries on average rated their work as moderately to considerably stressful. In both countries, the average response to statements relating to positive job attributes clustered around 'neither agree nor disagree', and the average response to statements relating to negative job attributes clustered around 'agree'. Although Scottish GPs had less intention to reduce work participation than English, significant minorities in both countries intended to reduce their hours or other work participation in the next five years.

Strengths and limitations

For peer review only - http://bmjopen.bmj.com/site/about/guidelines.xhtml

This is the first study to compare the working lives of GPs in Scotland and England using a set of common, validated questions on satisfaction, job characteristics and job pressures and using a similar data collection method. In both countries, largely nationally-representative samples were collected. We have adjusted the differences for respondents' age, gender, ethnicity and partner status, as well as the deprivation and urbanicity of the populations registered with the practices at which these GPs work.

The response rate was lower to the England survey. Evidence suggests that less satisfied GPs are more likely to respond to job satisfaction surveys [24]. If also true in this context, then the lower response rate in the English survey may mean it represents a pool of less satisfied English GPs and the differences could overestimate the true population differences. However, our supplementary analyses restricting the Scottish sample to the first 25.2% of returned surveys to match the total response rate in the English survey, showed only trivial differences compared to the full sample (see Supplementary file Tables S6 and S7).

Comparison with existing literature

The current 2017 survey in England work builds on previous English surveys on GP satisfaction using the same validated measures. This is the largest study ever on GPs' views on working lives in Scotland, and the first major study since the new Scottish GP contract was introduced in April 2018. Although Scotland generally had better scores than England, many of the absolute values in Scotland still give cause for concern. Almost a quarter (23.8%) of GPs in Scotland rated their chance of leaving direct patient care as 'considerable' or 'high'. This compares with a small survey conducted by RCGP Scotland in 2018 which found 26% of GPs felt they would be unlikely to be working in general practice in the next five years [25]. Thus, 'better' than England, does not necessarily mean 'good', and the findings give no room for complacency in either country.

Implications for research and policy

For peer review only - http://bmjopen.bmj.com/site/about/guidelines.xhtml

We assume (at least some of) the differences between GPs in Scotland and England in the present surveys were due to the timing and contract differences. Responsiveness of job satisfaction to new contracts has been shown before in the UK[26], although it is conceivable that differences between the two countries may have existed prior to the more recent policy changes; new Scottish contract reflects longer-standing differences from England in relation to NHS organisation, performance management and professional-managerial relationships [12].

In England, since the current survey was carried out, a new contract has been introduced in 2019 which focuses upon so-called 'Primary Care Networks' (PCNs) [27]. These are intended to cover between 30-50,000 people, although some are considerably larger[28]. The English contract also provides direct reimbursement for non-GP practitioners (e.g., pharmacists, physiotherapists, etc.) working within the PCN; payment for participating in a PCN; and access to incentive funding. Practices are required to deliver structured medication reviews, more support for Care Homes, early diagnosis of cancer, and anticipatory care for those most at risk of hospital admission. It is not yet clear what effect these changes will have on GPs' workload and job satisfaction.[29] The initial draft of the service specifications [30] was rejected by GPs as a significant burden of extra work [31,32].

Our study suggests that GPs in England feel additional pressures across all domains of practice compared to their Scottish colleagues. Whilst working together collaboratively as a PCN may increase satisfaction by engendering a sense of local community and support, it seems likely that overall satisfaction will only increase if perceived negative job attributes and job stressors also reduce. It is therefore of vital importance to understand how the recruitment of non-GPs into general practice impacts upon different types of work.

The COVID-19 crisis has clearly had a significant impact on general practice in both countries, with practices switching to a 'triage first' model with markedly reduced face-to-face contact with

For peer review only - http://bmjopen.bmj.com/site/about/guidelines.xhtml

patients.[33] Some of these changes may endure, but it is not at present clear which. It will be some time before we can understand the impact of COVID-19 on GP work, satisfaction and retention.

Further research is required to look at trends over time, as primary care reforms proceed across the UK in the coming years, and as the impact of COVID-19 evolves. In addition, research is required to understand how different elements of contract changes impact particular domains of satisfaction, and how workload changes in response to particular innovations.

For peer review only

Acknowledgements

We would like to thank all the GPs in England and Scotland who took part in this study.

Funding

The GP worklife survey in England was funded by the NIHR Policy Research Programme Policy Research Unit in the Health and Care System and Commissioning. The views expressed in the publication are those of the author(s) and not necessarily those of the NHS, the NIHR, the Department of Health and Social Care, arm's length bodies or other government departments. The GP worklife survey in Scotland was funded by the Scottish School of Primary Care.

Author contributions

For peer review only - http://bmjopen.bmj.com/site/about/guidelines.xhtml

English survey: Matt Sutton and Kath Checkland devised the project. Helen Hayes, Matt Sutton and Jon Gibson were involved in the planning and design of methods. Jon Gibson, Kath Checkland and Matt Sutton were involved in the data collection for the English data. The data cleaning and analysis was carried out by Helen Hayes. Helen Hayes, Matt Sutton, Jon Gibson and Kath Checkland were involved in the writing of the manuscript.

Scottish survey: Stewart Mercer, Bruce Guthrie, John Gillies, and Bridie Fitzpatrick devised the project. Bruce Guthrie, Stewart Mercer, John Gillies, and Bridie Fitzpatrick were involved in the planning and design of the methods. Bridie Fitzpatrick led the data collection and data cleaning. Helen Hayes carried out the data analysis. Stewart Mercer led the revisions to the manuscript with input from Bruce Guthrie. Bridie Fitzpatrick and John Gillies read and revised the later versions.

All authors read and agreed the final version prior to submission. The corresponding author attests that all listed authors meet authorship criteria and that no others meeting the criteria have been omitted.

No competing interests

All authors have completed the ICMJE uniform disclosure form at www.icmje.org/coi_disclosure.pdf and declare: no support from any organisation for the submitted work; no financial relationships with any organisations that might have an interest in the submitted work in the previous three years; no other relationships or activities that could appear to have influenced the submitted work.

Patient and Public Involvement

There was no patient and public involvement in this study.

For peer review only - http://bmjopen.bmj.com/site/about/guidelines.xhtml

Dissemination declaration

We presented the findings of the study at the Scottish School of Primary Care conference on the 7th of May 2019.

Bibliography

1. Pathman DE, Konrad TR, Williams ES, et al. Physician Job satisfaction, job dissatisfaction, and physician turnover. J Fam Pract. Vol. 51. 2002.

2. Scott A, Gravelle H, Simoens S, et al. Job satisfaction and quitting intentions: A structural model of British general practitioners. Br J Ind Relations. 2006 Sep;44(3):519–40.

3. Panagioti M, Geraghty K, Johnson J, et al. Association Between Physician Burnout and Patient Safety, Professionalism, and Patient Satisfaction. JAMA Intern Med [Internet]. 2018 Oct 1 [cited 2020 May 26];178(10):1317. Available from: https://jamanetwork.com/journals/jamainternalmedicine/fullarticle/2698144

4. West CP, Dyrbye LN, Shanafelt TD. Physician burnout: contributors, consequences and solutions. Vol. 283, Journal of Internal Medicine. Blackwell Publishing Ltd; 2018. p. 516–29.

5. Martin S, Davies E, Gershlick B. Under Pressure: What the Commonwealth Fund's 2015 International Survey of General Practitioners Means for the UK [Internet]. Analysis. 2016 [cited 2020 May 26]. Available from: http://www.health.org.uk/sites/health/files/UnderPressure.pdf

6. Fisher R, Turton C, Gershlick B et al. Feeling the strain: What The Commonwealth Fund's 2019

For peer review only - http://bmjopen.bmj.com/site/about/guidelines.xhtml

international survey of general practitioners means for the UK. The Health Foundation. 2020.

7. Roland M, Everington S. Tackling the crisis in general practice: If general practice fails, the whole NHS fails. Vol. 352, BMJ (Online). BMJ Publishing Group; 2016.

8. Hobbs FDR, Bankhead C, Mukhtar T, et al. Clinical workload in UK primary care: a retrospective analysis of 100 million consultations in England, 2007–14. Lancet [Internet]. 2016 [cited 2020 May 26];387(10035):2323–30. Available from: https://www.sciencedirect.com/science/article/pii/S0140673616006206

9. Gibson J, Sutton M, Spooner S, Checkland K. Ninth National GP Worklife Survey. University of Manchester: Policy Research Unit in Commissioning and the Healthcare System Manchester Centre for Health Economics; 2018. p. 36 p.

10. NHS Digital. General Practice Workforce, Final 30 September 2018, Experimental statistics [Internet]. 2018 [cited 2020 May 26]. Available from: https://digital.nhs.uk/data-and-information/publications/statistical/general-practice-workforce-archive/final-30-september-2018-experimental-statistics

11. Smith GI, Mercer SW, Gillies JCM, McDevitt A. Improving together: A new quality framework for GP clusters in Scotland. Vol. 67, British Journal of General Practice. Royal College of General Practitioners; 2017. p. 294–5.

12. The Scottish Government and BMA. The 2018 General Medical Services Contract in Scotland [Internet]. 2017 [cited 2020 May 26]. Available from: https://www.gov.scot/publications/gms-contract-scotland/

13. Warr P, Cook J, Wall T. Scales for the measurement of some work attitudes and aspects of psychological well-being. J Occup Psychol. 1979 Jun 1;52(2):129–48.

14. Sibbald B, Bojke C, Gravelle H. National survey of job satisfaction and retirement intentions among general practitioners in England. Br Med J. 2003 Jan 4;326(7379):22–4.

15. Mercer S. GP Survey - Scottish Findings. 2019.

For peer review only - http://bmjopen.bmj.com/site/about/guidelines.xhtml

16. ONS. 2011 rural/urban classification [Internet]. 2016 [cited 2020 May 26]. Available from: https://www.ons.gov.uk/methodology/geography/geographicalproducts/ruralurbanclassifications/2011ruralurbanclassification

17. Abel GA, Barclay ME, Payne RA. Adjusted indices of multiple deprivation to enable comparisons within and between constituent countries of the UK including an illustration using mortality rates. BMJ Open [Internet]. 2016 [cited 2020 May 26];6(11). Available from: http://bmjopen.bmj.com/

18. NHS Digital. General and Personal Medical Services, England As at 31 December 2017, Provisional Experimental statistics [Internet]. 2018 [cited 2020 May 26]. Available from: https://digital.nhs.uk/data-and-information/publications/statistical/general-practice-workforce-archive/as-at-31-december-2017-provisional-experimental-statistics

19. ISD Scotland. General Practice - GP Workforce and practice list sizes 2008-2018 [Internet]. 2018 [cited 2020 May 26]. Available from: https://www.isdscotland.org/Health-Topics/General-Practice/Publications/2018-12-11/2018-12-11-GPWorkforce2018-Report.pdf

20. ISD Scotland. List of GPs in post and their practice contact details [Internet]. 2019. Available from: https://www.isdscotland.org/Health-Topics/General-Practice/Workforce-and-Practice-Populations/

21. Abel G, Payne R, Barclay M. UK Deprivation Indices. University of Bristol; 2016.

22. NHS Digital. Patients Registered at a GP Practice, January 2018; Special Topic - Patients age group comparison [Internet]. 2018 [cited 2020 May 26]. Available from: https://digital.nhs.uk/data-and-information/publications/statistical/patients-registered-at-a-gp-practice/january-2018

23. Roland M, Guthrie B. Quality and Outcomes Framework: What have we learnt? BMJ. 2016 Aug 4;354.

24. Gravelle H, Hole AR, Hossain MI. Response bias in job satisfaction surveys: English general practitioners. Discussion papers in economics, no. 2008/24; 2008.

For peer review only - http://bmjopen.bmj.com/site/about/guidelines.xhtml

25. RCGP Scotland. From the Frontline - The changing landscape of Scottish general practice. 2019.

26. Whalley D, Gravelle H, Sibbald B. Effect of the new contract on GPs' working lives and perceptions of quality of care: A longitudinal survey. Br J Gen Pract. 2008 Jan 1;58(546):8–14.

27. NHS England, BMA. Investment and evolution: A five-year framework for GP contract reform to implement The NHS Long Term Plan. 2019;

28. Morciano M, Checkland K, Hammond J, et al. Variability in size and characteristics of England's Primary Care Networks: observational study [Internet]. British Journal of General Practice; 2020 [cited 2020 May 29]. Available from: https://www.research.manchester.ac.uk/portal/en/publications/variability-in-size-and-characteristics-of-englands-primary-care-networks-observational-study(4f9596ac-3fdd-4f05-82a2-b61893733601).html

29. Sibbald B, Shen J, McBride A. Changing the skill-mix of the health care workforce. J Health Serv Res Policy [Internet]. 2004 Jan [cited 2020 Jan 19];9 Suppl 1:28–38. Available from: http://www.ncbi.nlm.nih.gov/pubmed/15006226

30. NHS England and NHS Improvement. Network Contract Direct Enhanced Service - Draft Service Specifications. 2019.

31. Murray R. GPs condemn new specifications for primary care networks. Vol. 368, The BMJ. BMJ Publishing Group; 2020.

32. Wilson T, Lewis R. Primary care networks: Well intentioned but overambitious. Vol. 366, The BMJ. BMJ Publishing Group; 2019.

33. NHS England, NHS Improvement. Guidance and standard operating procedures: General practice in the context of coronavirus (COVID-19). 2020.

For peer review only - http://bmjopen.bmj.com/site/about/guidelines.xhtml

For peer review only

Table 1: Differences between Scotland and England in intentions to reduce work participation

	Scotland (Mean, SD)	England (Mean, SD)	Unadjusted difference (Coef., 95% CI)	Adjusted difference (Coef., 95% CI)
Reduce your work hours within five years?	2.78 (1.56)	3.54 (1.49)	-0.76 [-0.88 to -0.64]	-0.52 [-0.64 to -0.41]
Leave medical work entirely within five years?	2.11 (1.49)	2.76 (1.63)	-0.65 [-0.78 to -0.52]	-0.32 [-0.42 to -0.22]
Leave direct patient care within five years?	2.23 (1.51)	2.93 (1.59)	-0.70 [-0.82 to -0.58]	-0.37 [-0.47 to -0.27]
Continue with medical work but outside the UK within five years?	1.41 (0.84)	1.61 (1.03)	-0.21 [-0.28 to -0.13]	-0.20 [-0.28 to -0.12]

Notes: N=2,927. All four measures of intentions to reduce work participation are measured on a five-point scale, 1=”none” to 5=”high”. Columns 4 and 5 are coefficients from linear regressions with heteroscedasticity-robust standard errors. Adjusted differences control for gender and age category interactions, ethnicity, partner status, and practice deprivation and urbanicity.

Table 2: Differences between Scotland and England in satisfaction, job stressors and job attributes

	Scotland (Mean, SD)	England (Mean, SD)	Unadjusted difference (Coef., 95% CI)	Adjusted difference (Coef., 95% CI)
Satisfaction	5.27 (0.97)	4.71 (1.07)	0.55 [0.47 to 0.63]	0.50 [0.42 to 0.59]
Job stressors	3.53 (0.75)	3.86 (0.66)	-0.34 [-0.39 to -0.28]	-0.29 [-0.35 to -0.23]
Positive job attributes	3.19 (0.57)	3.15 (0.62)	0.037 [-0.011 to 0.085]	0.013 [-0.038 to 0.063]
Negative job attributes	4.03 (0.64)	4.30 (0.57)	-0.27 [-0.32 to -0.23]	-0.27 [-0.31 to -0.22]

Notes: N=2,927. Satisfaction is measured 1 (extremely dissatisfied) to 7 (extremely satisfied). Job stressors are measured 1 (no pressure) to 5 (high pressure). Job attributes are measured 1 (strongly disagree) to 5 (strongly agree) with 3 neutral. Columns 4 and 5 are coefficients from linear regressions with robust standard errors. Adjusted differences control for age, gender, age x gender interaction, ethnicity, partner, and practice deprivation and urbanicity.

For peer review only - http://bmjopen.bmj.com/site/about/guidelines.xhtml

For peer review only

For peer review only - http://bmjopen.bmj.com/site/about/guidelines.xhtml

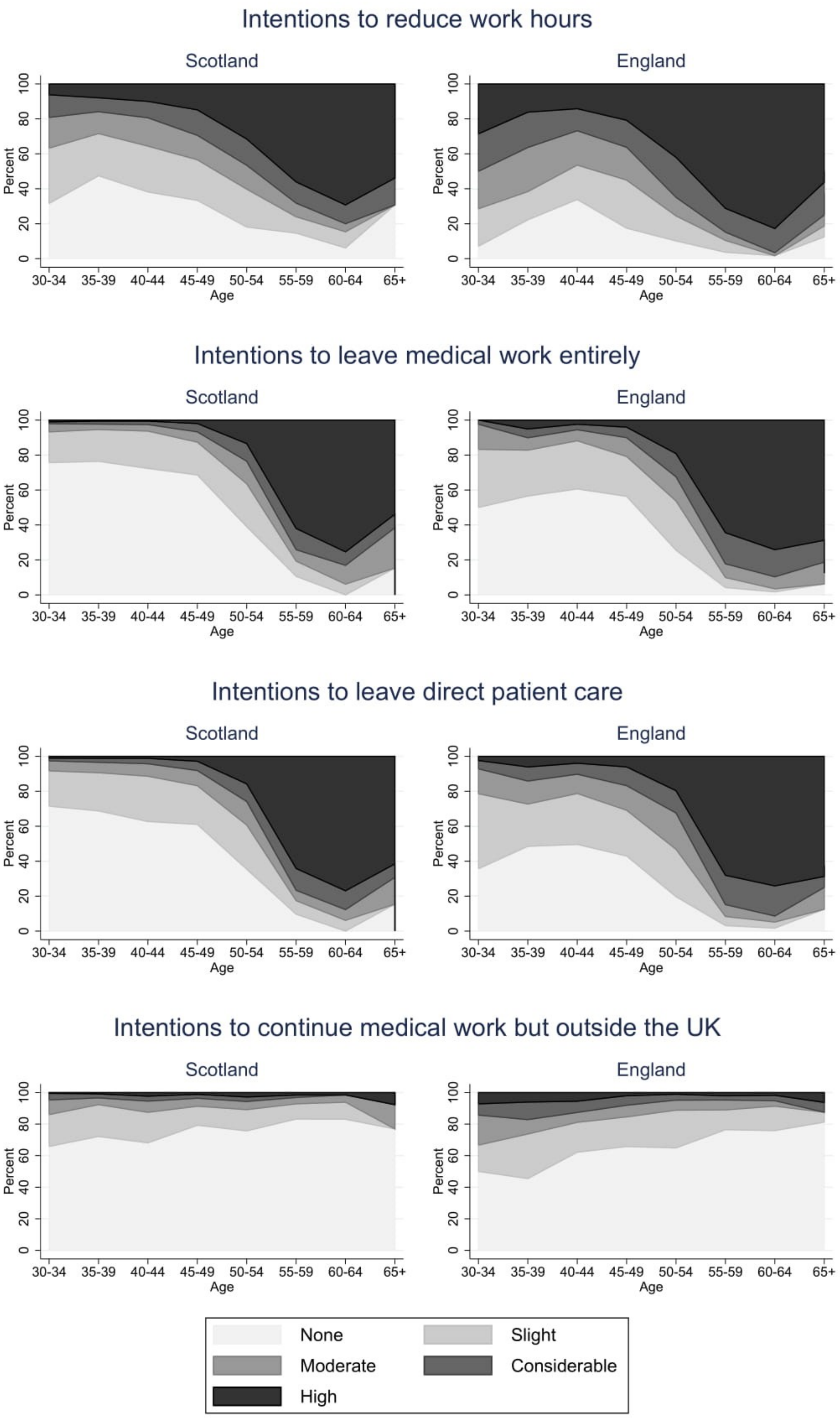
Intentions to reduce work hours
Scotland
England
Intentions to leave medical work entirely
Scotland
England
Intentions to leave direct patient care
Scotland
England
Intentions to continue medical work but outside the UK
Scotland
England
Percent
0
20
40
60
80
100
Age
30-34
35-39
40-44
45-49
50-54
55-59
60-64
65+
None
Slight
Moderate
Considerable
High

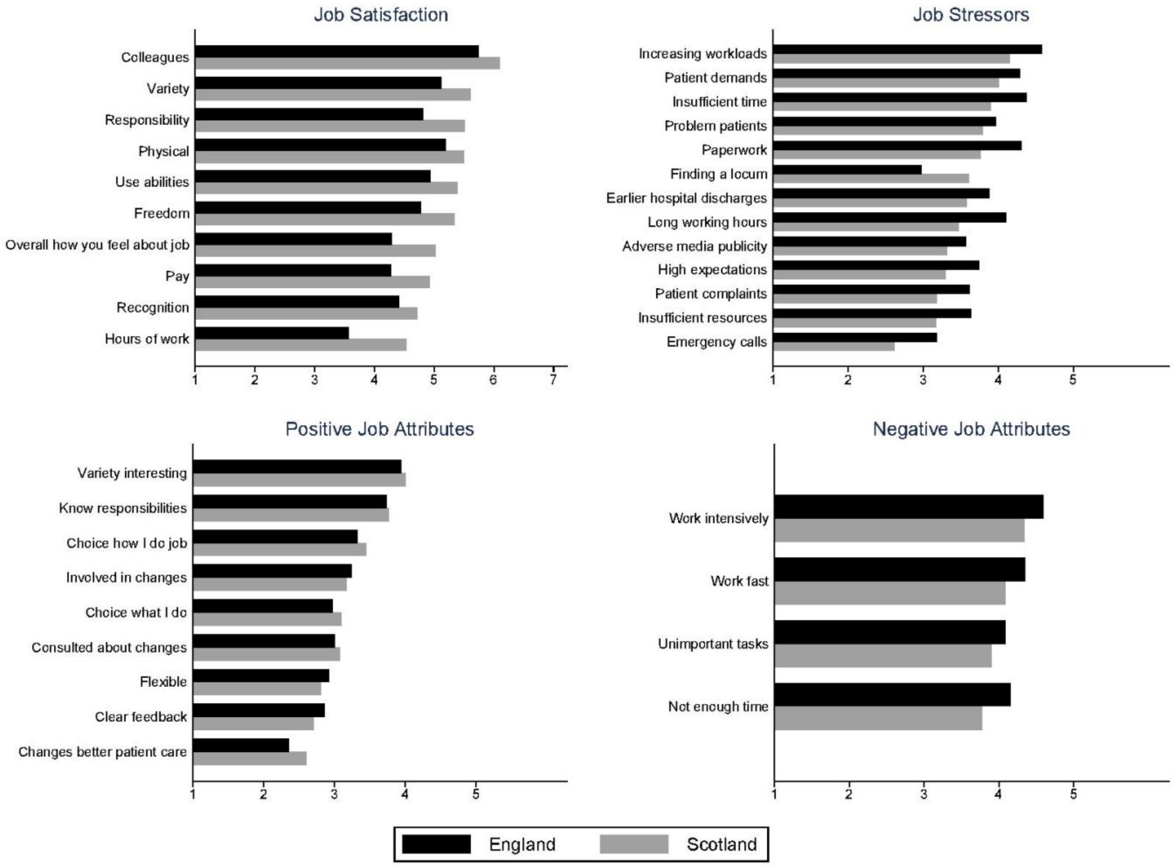

For peer review only

SUPPLEMENTARY FILE

For peer review only

Table S1: Country differences in each domain of job satisfaction

Job satisfaction domain	Scotland (Mean, SD)	England (Mean, SD)	Unadjusted difference (Coeff, CI)	Adjusted difference (Coeff, CI)
Your colleagues and fellow workers	6.10 (1.14)	5.75 (1.23)	0.35 [0.26 to 0.45]	0.30 [0.20 to 0.39]
Amount of variety in your job	5.61 (1.20)	5.12 (1.38)	0.49 [0.39 to 0.60]	0.44 [0.34 to 0.55]
Amount of responsibility you are given	5.51 (1.43)	4.81 (1.68)	0.70 [0.57 to 0.83]	0.61 [0.48 to 0.74]
Physical working conditions	5.50 (1.45)	5.19 (1.45)	0.31 [0.19 to 0.42]	0.29 [0.18 to 0.41]
Opportunities to use your abilities	5.38 (1.26)	4.93 (1.45)	0.45 [0.34 to 0.56]	0.40 [0.29 to 0.52]
Freedom to choose your own method of working	5.34 (1.27)	4.78 (1.45)	0.56 [0.45 to 0.67]	0.53 [0.41 to 0.64]
Taking everything into consideration, how do you feel about your job	5.02 (1.34)	4.29 (1.49)	0.73 [0.62 to 0.85]	0.66 [0.54 to 0.78]
Your remuneration	4.93 (1.57)	4.28 (1.65)	0.65 [0.53 to 0.78]	0.60 [0.47 to 0.74]
Recognition you get for good work	4.72 (1.54)	4.41 (1.58)	0.31 [0.19 to 0.44]	0.28 [0.15 to 0.41]
Your hours of work	4.53 (1.66)	3.57 (1.79)	0.96 [0.82 to 1.10]	0.88 [0.74 to 1.03]

Notes: N=2,927. Satisfaction components are measured on a seven-point scale. Columns 4 and 5 are coefficients from linear regressions with robust standard errors. Adjusted differences control for age, gender, age x gender interaction, ethnicity, partner, and practice deprivation and urbanicity.

For peer review only

Table S2: Country differences in each job stressor

Job stressor	Scotland (Mean, SD)	England (Mean, SD)	Unadjusted difference (Coeff, CI)	Adjusted difference (Coeff, CI)
Increasing workloads	4.15 (0.93)	4.58 (0.68)	-0.42 [-0.48 to -0.36]	-0.41 [-0.47 to -0.35]
Increased demands from patients	4.01 (0.91)	4.29 (0.73)	-0.28 [-0.34 to -0.22]	-0.27 [-0.34 to -0.21]
Having insufficient time to do justice to the job	3.90 (1.04)	4.38 (0.83)	-0.48 [-0.55 to -0.41]	-0.47 [-0.54 to -0.39]
Dealing with problem patients	3.79 (0.97)	3.97 (0.88)	-0.17 [-0.24 to -0.10]	-0.16 [-0.24 to -0.084]
Paperwork	3.77 (0.97)	4.31 (0.87)	-0.54 [-0.62 to -0.47]	-0.52 [-0.59 to -0.44]
Finding a locum	3.61 (1.42)	2.98 (1.41)	0.63 [0.52 to 0.74]	0.75 [0.64 to 0.86]
Dealing with earlier discharges from hospital	3.58 (0.99)	3.88 (0.93)	-0.30 [-0.37 to -0.22]	-0.24 [-0.32 to -0.16]
Long working hours	3.47 (1.16)	4.10 (1.04)	-0.63 [-0.72 to -0.55]	-0.59 [-0.68 to -0.50]
Adverse publicity by the media	3.32 (1.25)	3.57 (1.22)	-0.25 [-0.34 to -0.15]	-0.21 [-0.31 to -0.11]
Unrealistically high expectation of role by others	3.30 (1.20)	3.75 (1.12)	-0.45 [-0.54 to -0.36]	-0.38 [-0.48 to -0.29]
Worrying about patient complaints/litigation	3.18 (1.13)	3.61 (1.12)	-0.44 [-0.52 to -0.35]	-0.38 [-0.48 to -0.29]
Insufficient resources within the practice	3.17 (1.16)	3.64 (1.15)	-0.47 [-0.56 to -0.38]	-0.38 [-0.47 to -0.28]
Interruptions by emergency calls during surgery	2.62 (1.10)	3.18 (1.11)	-0.57 [-0.66 to -0.48]	-0.52 [-0.61 to -0.43]

Notes: N=2,927. Stressors components are measured on a five-point scale. Columns 4 and 5 are coefficients from linear regressions with robust standard errors. Adjusted differences control for age, gender, age x gender interaction, ethnicity, partner, and practice deprivation and urbanicity.

For peer review only

Table S3: Country differences in each positive job attribute

Positive job attribute	Scotland (Mean, SD)	England (Mean, SD)	Unadjusted difference (Coeff, CI)	Adjusted difference (Coeff, CI)
My job provides me with a variety of interesting things	4.01 (0.73)	3.95 (0.78)	0.059 [-0.0019 to 0.12]	0.032 [-0.029 to 0.093]
I always know what my work responsibilities are	3.77 (0.85)	3.74 (0.93)	0.034 [-0.037 to 0.11]	0.0022 [-0.073 to 0.077]
I have a choice in deciding how I do my job	3.45 (0.94)	3.32 (1.00)	0.13 [0.051 to 0.21]	0.091 [0.011 to 0.17]
I am involved in deciding on the changes introduced that affect my work	3.17 (1.10)	3.24 (1.22)	-0.067 [-0.16 to 0.027]	-0.088 [-0.18 to 0.0088]
I have a choice in deciding what I do at work	3.10 (0.99)	2.97 (1.01)	0.13 [0.052 to 0.21]	0.10 [0.021 to 0.19]
I am consulted about changes that affect my work	3.08 (1.10)	3.01 (1.28)	0.072 [-0.025 to 0.17]	0.0052 [-0.096 to 0.11]
My working time can be flexible	2.81 (1.06)	2.92 (1.05)	-0.11 [-0.19 to -0.026]	-0.12 [-0.21 to -0.037]
I get clear feedback about how well I am doing my job	2.70 (0.92)	2.86 (0.96)	-0.16 [-0.23 to -0.082]	-0.13 [-0.21 to -0.049]
Changes to my job in the last year have led to better patient care	2.60 (0.95)	2.36 (0.97)	0.24 [0.17 to 0.32]	0.22 [0.14 to 0.30]

Notes: N=2,927. Positive job attributes components are measured on a five-point scale. Columns 4 and 5 are coefficients from linear regressions with robust standard errors. Adjusted differences control for age, gender, age x gender interaction, ethnicity, partner, and practice deprivation and urbanicity.

For peer review only

Table S4: Country differences in each negative job attribute

Negative job attributes domain	Scotland (Mean, SD)	England (Mean, SD)	Unadjusted difference (Coeff, CI)	Adjusted difference (Coeff, CI)
I have to work very intensively	4.34 (0.73)	4.60 (0.62)	-0.26 [-0.31 to -0.21]	-0.26 [-0.32 to -0.21]
I have to work very fast	4.09 (0.83)	4.35 (0.76)	-0.26 [-0.33 to -0.20]	-0.26 [-0.33 to -0.20]
I am required to do unimportant tasks which prevent me completing more important ones	3.90 (0.97)	4.09 (0.97)	-0.19 [-0.27 to -0.11]	-0.18 [-0.26 to -0.10]
I do not have time to carry out all my work	3.78 (1.02)	4.16 (0.98)	-0.38 [-0.46 to -0.30]	-0.36 [-0.44 to -0.28]

Notes: N=2,927. Negative job attributes components are measured on a five-point scale. Columns 4 and 5 are coefficients from linear regressions with robust standard errors. Adjusted differences control for age, gender, age x gender interaction, ethnicity, partner, and practice deprivation and urbanicity.

For peer review only

Table S5: Summary statistics for the covariates showing representativeness of the two surveys

Variable names	Scotland: Survey GPs (n=2,048) %	Scotland: All GPs (Sep 2018) (n=4,366) %	England: Survey GPs (n=879) %	England: All GPs (Dec 2017) (n=34,549 %
Female	58%	58%	51%	54%
GP Partner	84%	78%	87%	66%
Black, Asian and Minority Ethnicity	9%	n/a	20%	n/a
Age category:				
<35 years	10%	13%	5%	12%
35-44 years	34%	34%	26%	34%
45-54 years	36%	33%	38%	31%
55-64 years	19%	18%	28%	19%
65+ years	1%	1%	3%	4%
Proportion of patients in an urban area	65%	66%	77%	81%
Proportion of patients in most deprived quintile	20%	19%	16%	19%

Notes: All data are presented for salaried and partnered GPs only. This is with the exception of population figures for deprivation and urbanicity indicators for Scotland, as GP headcount data by practice used to create this figure is only available for all GPs (including registrars, retainers and locums).
For deprivation, we use data on the number of patients living in each deprivation quintile registered with each practice. For Scotland, number of patients in each deprivation quintile is publicly available. For England, this is created using data on LSOA level quintiles of IMD and the number of patients in each LSOA.
For urbanicity, we use the distribution of patients in urban and rural areas. For Scotland, number of patients in each practice in each urban/rural classification is publicly available. For England this was created using the 2011 urban/rural classification by LSOA and the number of patients in each practice in each LSOA.
Ethnicity data is unavailable at the population level.

For peer review only

Table S6: Restricted sample analysis of differences between Scotland and England in intentions to reduce work participation

	Scotland (Mean, SD)	England (Mean, SD)	Unadjusted difference (Coef., 95% CI)	Adjusted difference (Coef., 95% CI)
Reduce your work hours within five years?	2.84 (1.58)	3.54 (1.49)	-0.70 [-0.84 to -0.55]	-0.53 [-0.66 to -0.39]
Leave medical work entirely within five years?	2.20 (1.53)	2.76 (1.63)	-0.55 [-0.70 to -0.41]	-0.31 [-0.42 to -0.20]
Leave direct patient care within five years?	2.33 (1.54)	2.93 (1.59)	-0.60 [-0.74 to -0.45]	-0.36 [-0.47 to -0.24]
Continue with medical work but outside the UK within five years?	1.37 (0.79)	1.61 (1.03)	-0.25 [-0.33 to -0.16]	-0.22 [-0.30 to -0.13]

Notes: N=1,800. Intentions to reduce work participation indicators are measured on a five-point scale. Columns 4 and 5 are coefficients from linear regressions with robust standard errors. Adjusted differences control for age, gender, age x gender interaction, ethnicity, partner, and practice deprivation and urbanicity.
Analysis is based on the first 25.2% of respondents in the Scottish sample to match the returned English sample.

For peer review only

Table S7: Restricted sample analysis of differences between Scotland and England across all satisfaction, job stressors, and job attributes domains

	Scotland (Mean, SD)	England (Mean, SD)	Unadjusted difference (Coef., 95% CI)	Adjusted difference (Coef., 95% CI)
Satisfaction	5.29 (0.98)	4.71 (1.07)	0.58 [0.49 to 0.68]	0.51 [0.41 to 0.61]
Job stressors	3.53 (0.78)	3.86 (0.66)	-0.33 [-0.40 to -0.26]	-0.28 [-0.35 to -0.21]
Positive job attributes	3.19 (0.60)	3.15 (0.62)	0.037 [-0.020 to 0.093]	0.0054 [-0.053 to 0.064]
Negative job attributes	4.01 (0.66)	4.30 (0.57)	-0.29 [-0.35 to -0.23]	-0.28 [-0.34 to -0.22]

Notes: N=1,800. Satisfaction is measured 1-7. Job stressors and job attributes are measured 1-5. Columns 4 and 5 are coefficients from linear regressions with robust standard errors. Adjusted differences control for age, gender, age x gender interaction, ethnicity, partner, and practice deprivation and urbanicity.
Analysis is based on the first 25.2% of respondents in the Scottish sample to match the returned English sample.

For peer review only

STROBE Statement—Checklist of items that should be included in reports of ***cross-sectional studies***

	Item No	Recommendation	Page No
**Title and abstract**	1	(*a*) Indicate the study's design with a commonly used term in the title or the abstract	1
		(*b*) Provide in the abstract an informative and balanced summary of what was done and what was found	2,3
**Introduction**			
Background/rationale	2	Explain the scientific background and rationale for the investigation being reported	4,5
Objectives	3	State specific objectives, including any prespecified hypotheses	5
**Methods**			
Study design	4	Present key elements of study design early in the paper	7
Setting	5	Describe the setting, locations, and relevant dates, including periods of recruitment, exposure, follow-up, and data collection	7
Participants	6	(*a*) Give the eligibility criteria, and the sources and methods of selection of participants	7
Variables	7	Clearly define all outcomes, exposures, predictors, potential confounders, and effect modifiers. Give diagnostic criteria, if applicable	7,8
Data sources/ measurement	8*	For each variable of interest, give sources of data and details of methods of assessment (measurement). Describe comparability of assessment methods if there is more than one group	7,8
Bias	9	Describe any efforts to address potential sources of bias	9
Study size	10	Explain how the study size was arrived at	6,7
Quantitative variables	11	Explain how quantitative variables were handled in the analyses. If applicable, describe which groupings were chosen and why	9
Statistical methods	12	(*a*) Describe all statistical methods, including those used to control for confounding	9
		(*b*) Describe any methods used to examine subgroups and interactions	9
		(*c*) Explain how missing data were addressed	9
		(*d*) If applicable, describe analytical methods taking account of sampling strategy	9
		(*e*) Describe any sensitivity analyses	
**Results**			
Participants	13*	(a) Report numbers of individuals at each stage of study—eg numbers potentially eligible, examined for eligibility, confirmed eligible, included in the study, completing follow-up, and analysed	Suppl. File
		(b) Give reasons for non-participation at each stage	N/A
		(c) Consider use of a flow diagram	N/A
Descriptive data	14*	(a) Give characteristics of study participants (eg demographic, clinical, social) and information on exposures and potential confounders	Suppl. File
		(b) Indicate number of participants with missing data for each variable of interest	7
Outcome data	15*	Report numbers of outcome events or summary measures	10
Main results	16	(*a*) Give unadjusted estimates and, if applicable, confounder-adjusted estimates and their precision (eg, 95% confidence interval). Make clear which confounders were adjusted for and why they were included	10,11 +

For peer review only

			Suppl. File
		(*b*) Report category boundaries when continuous variables were categorized	10,11
		(*c*) If relevant, consider translating estimates of relative risk into absolute risk for a meaningful time period	N/A
Other analyses	17	Report other analyses done—eg analyses of subgroups and interactions, and sensitivity analyses	10,11
**Discussion**			
Key results	18	Summarise key results with reference to study objectives	12
Limitations	19	Discuss limitations of the study, taking into account sources of potential bias or imprecision. Discuss both direction and magnitude of any potential bias	12,13
Interpretation	20	Give a cautious overall interpretation of results considering objectives, limitations, multiplicity of analyses, results from similar studies, and other relevant evidence	13-15
Generalisability	21	Discuss the generalisability (external validity) of the study results	14,15
**Other information**			
Funding	22	Give the source of funding and the role of the funders for the present study and, if applicable, for the original study on which the present article is based	16

*Give information separately for exposed and unexposed groups.

Note: An Explanation and Elaboration article discusses each checklist item and gives methodological background and published examples of transparent reporting. The STROBE checklist is best used in conjunction with this article (freely available on the Web sites of PLoS Medicine at http://www.plosmedicine.org/, Annals of Internal Medicine at http://www.annals.org/, and Epidemiology at http://www.epidem.com/). Information on the STROBE Initiative is available at www.strobe-statement.org.

For peer review only

BMJ Open

Working lives of GPs in Scotland and England: cross-sectional analysis of national surveys

Journal:	*BMJ Open*
Manuscript ID	bmjopen-2020-042236.R1
Article Type:	Original research
Date Submitted by the Author:	23-Sep-2020
Complete List of Authors:	Hayes, Helen; The University of Manchester<br>Gibson, Jonathan; University of Manchester,<br>Fitzpatrick, Bridie; University of Glasgow, Institute of Health and Wellbeing<br>Checkland, Kath; The University of Manchester, Institute of Population Health; Centre for Biostatistics<br>Guthrie, Bruce; University of Edinburgh College of Medicine and Veterinary Medicine,<br>Sutton, Matt; The University of Manchester<br>Gillies, John; The University of Edinburgh, The Usher Institute of Population Health Sciences and Informatics<br>Mercer, Stewart; The University of Edinburgh, The Usher Institute of Population Health Sciences and Informatics
**Primary Subject Heading**:	General practice / Family practice
Secondary Subject Heading:	Health policy
Keywords:	PRIMARY CARE, Health policy < HEALTH SERVICES ADMINISTRATION & MANAGEMENT, Organisation of health services < HEALTH SERVICES ADMINISTRATION & MANAGEMENT

SCHOLARONE™
Manuscripts

I, the Submitting Author has the right to grant and does grant on behalf of all authors of the Work (as defined in the below author licence), an exclusive licence and/or a non-exclusive licence for contributions from authors who are: i) UK Crown employees; ii) where BMJ has agreed a CC-BY licence shall apply, and/or iii) in accordance with the terms applicable for US Federal Government officers or employees acting as part of their official duties; on a worldwide, perpetual, irrevocable, royalty-free basis to BMJ Publishing Group Ltd ("BMJ") its licensees and where the relevant Journal is co-owned by BMJ to the co-owners of the Journal, to publish the Work in this journal and any other BMJ products and to exploit all rights, as set out in our licence.

The Submitting Author accepts and understands that any supply made under these terms is made by BMJ to the Submitting Author unless you are acting as an employee on behalf of your employer or a postgraduate student of an affiliated institution which is paying any applicable article publishing charge ("APC") for Open Access articles. Where the Submitting Author wishes to make the Work available on an Open Access basis (and intends to pay the relevant APC), the terms of reuse of such Open Access shall be governed by a Creative Commons licence – details of these licences and which Creative Commons licence will apply to this Work are set out in our licence referred to above.

Other than as permitted in any relevant BMJ Author's Self Archiving Policies, I confirm this Work has not been accepted for publication elsewhere, is not being considered for publication elsewhere and does not duplicate material already published. I confirm all authors consent to publication of this Work and authorise the granting of this licence.

For peer review only

Working lives of GPs in Scotland and England: cross-sectional analysis of national surveys

Ms Helen Hayes MA, MSc

Research Assistant

Division of Population Health, Health Services Research & Primary Care, University of Manchester, Oxford Road, Manchester M13 9PL

Dr Jonathon Gibson BA, PG Dip, MSc, PhD

Research Fellow

Division of Population Health, Health Services Research & Primary Care, University of Manchester, Oxford Road, Manchester M13 9PL

Dr Bridie Fitzpatrick MSc, PhD

Honorary Senior Research Fellow, Institute for Health and Wellbeing, University of Glasgow, Glasgow G12 9LX

Professor Katherine Checkland BMedSci, MBBS, MA, PhD, MRCGP

Professor of Health Policy and Primary Care, Division of Population Health, Health Services Research & Primary Care, University of Manchester, Oxford Road, Manchester M13 9PL

Professor Bruce Guthrie BA, MB BChir, MSc, PhD, MRCP, FRCGP, FRSE

Professor of General Practice, Centre for Populations Health Sciences, Usher Institute, University of Edinburgh, Teviot Place, Edinburgh EH8 9AG

Professor Mathew Sutton BA, MSc, PhD

Chair in Health Economics, Director of Applied Health Research Domain, Division of Population Health, Health Services Research & Primary Care, University of Manchester, Oxford Road, Manchester M13 9PL

Professor John Gillies OBE MBChB, MA, FRSE, FRCGP, FRCPE

Deputy Director of the Scottish School of Primary Care, Honorary Professor in General Practice, Centre for Populations Health Sciences, Usher Institute, University of Edinburgh, Teviot Place, Edinburgh EH8 9AG

Professor Stewart W Mercer BSc (Hons), MBChB, MSc, PhD, FRCGP, FRCPE, FFPHM*

Director of the Scottish School of Primary Care, Professor of Primary Care and Multimorbidity, Centre for Populations Health Sciences, Usher Institute, University of Edinburgh, Teviot Place, Edinburgh EH8 9AG

*Corresponding author: stewart.mercer@ed.ac.uk

For peer review only - http://bmjopen.bmj.com/site/about/guidelines.xhtml

Abstract

Objectives

The UK faces major problems retaining general practitioners (GPs). Scotland introduced a new GP contract in April 2018, intended to better support GPs. This study compares the career intentions and working lives of GPs in Scotland with GPs in England, shortly after the new Scotland contract was introduced.

Design and setting

Comparison of cross-sectional analysis of survey responses of GPs in England and Scotland in 2017 and 2018 respectively, using linear regression to adjust the differences for gender, age, ethnicity, urbanicity and deprivation.

Participants

2,048 GPs in Scotland and 879 GPs in England.

Main outcome measures

Four intentions to reduce work participation (five-point scales: 1='none', 5='high'): reducing working hours; leaving medical work entirely; leaving direct patient care; or continuing medical work but outside the UK. Four domains of working life: job satisfaction (seven-point scale: 1='extremely dissatisfied', 7='extremely satisfied'); job stressors (five point-scale: 1='no pressure', 5='high pressure); positive and negative job attributes (five-point scales: 1='strongly disagree', 5='strongly agree').

Results

Compared to England, GPs in Scotland had lower intentions to reduce work participation, including a lower likelihood of reducing work hours (2.78 versus 3.54; adjusted difference = -0.52; 95%CI -0.64 to -0.41), a lower likelihood of leaving medical work entirely (2.11 versus 2.76;

For peer review only

adjusted difference = -0.32; 95%CI -0.42 to -0.22), a lower likelihood of leaving direct patient care (2.23 versus 2.93; adjusted difference = -0.37; 95%CI -0.47 to -0.27), and a lower likelihood of continuing medical work but outside of the UK (1.41 versus 1.61; adjusted difference = -0.2; 95%CI -0.28 to -0.12). GPs in Scotland reported higher job satisfaction, lower job stressors, similar positive job attributes, and lower negative job attributes.

Conclusions

Following the introduction of the new contract in Scotland, GPs in Scotland reported significantly better working lives and lower intention to reduce work participation than England.

For peer review only

Strengths and Limitations

- This is the first study to compare the working lives of GPs in Scotland and England using a set of common, validated questions on satisfaction, job characteristics and job pressures and using a similar data collection method.
- In both countries, largely nationally-representative samples were collected.
- We have adjusted the differences for respondents' age, gender, ethnicity and partner status, as well as the deprivation and urbanicity of the populations registered with the practices at which these GPs work.
- The response rate was lower to the England survey. Evidence suggests that less satisfied GPs are more likely to respond to job satisfaction surveys 24. If also true in this context, then the lower response rate in the English survey may mean it represents a pool of less satisfied English GPs and the differences could overestimate the true population differences.
- However, our supplementary analyses restricting the Scottish sample to the first 25.2% of returned surveys to match the total response rate in the English survey, showed only trivial differences compared to the full sample

For peer review only

Introduction

Low physician job satisfaction, stress and burnout are associated with increased staff turnover and threats to patient safety[1–4] and contribute to recruitment and retention problems in general practice in the United Kingdom (UK). General Practitioners' (GPs) job satisfaction fell markedly in the UK between 2012 and 2015[5] and in a recent survey of 11 countries, UK GP job satisfaction was second lowest.[6] The general practice 'crisis'[7] in the UK reflects a substantial increase in workload[8] and surveys of English GPs' working lives have shown an increasing proportion intending to leave direct patient care within 5 years, up to a high of 39% in 2017.[9]

Despite policy ambitions to increase the number of GPs in the UK, there was a 1.4% decrease in full-time equivalent GPs in England between September 2017 and September 2018.[10] Failure to address GP recruitment and retention puts a number of health policy goals at risk, including shifting care out of hospital and into the community.

Whereas England has emphasised market mechanisms and inspection regimes to assure quality, Scotland has placed the emphasis on collaboration and integration. England retains the Quality and Outcomes Framework (QOF) and has implemented Care Quality Commission inspections. As the first step in negotiating and implementing a radical new GP contract in April 2018, Scotland abolished the QOF in 2016 and established GP Clusters as a clear signal of a shift to a more collaborative way of working. [11,12]

GP job satisfaction is an important indicator of the impact of GP contractual reform. We compare this between GPs in England in 2017 and Scotland in 2018 using cross-sectional survey data.

For peer review only - http://bmjopen.bmj.com/site/about/guidelines.xhtml

Methods

Design and setting

Data for England was from the 2017 National GP Worklife Survey,[9]using validated measures of satisfaction,[13]job pressure and attributes.[14] Data for Scotland was from the Scottish School of Primary Care survey and included the same validated measures.[15]

Ethical approval was granted for the English survey from the University of Manchester's Research and Ethics committee on the 5/10/2017 (Ref: 2017-2638-3884). The Scottish survey was assessed as not requiring ethical approval by the NHS or the University of Glasgow MVLS College Ethics Committee.

Participants

The English survey questionnaire was first posted to a random sample of 4,000 GPs in October 2017 with an option to complete an online version. Two reminders letters were sent in November and December 2017. Responses were returned by 996 GPs, representing 25.2% of the 3,953 eligible respondents after excluding GPs who had died, retired, left the practice or returned blank questionnaires.[9]

The Scottish survey questionnaire was first mailed to all 4,371 GPs in July 2018 which included the option of completing an online version, followed by two reminder paper mailings with further copies of the questionnaire in August and September 2018. Additionally, two further email reminders were sent (with the link to the online version) were sent mid and late August. The Scotland sample includes responses from 2,465 GPs (56.4% response rate). Due to the higher response rate, we ran supplementary analyses which restricted the sample in Scotland to the first 25.2% of returned surveys, to match the response rate in England.

The final sample consisted of 2,927 GPs (2,048 GPs in Scotland and 879 GPs in England) after removal of individuals with missing information on the outcome variables and covariates.

For peer review only - http://bmjopen.bmj.com/site/about/guidelines.xhtml

Outcome variables

We measured GPs' intentions to reduce work participation and four related domains of working lives; job satisfaction, job stressors, positive job attributes, and negative job attributes. For each domain, we created an average score for all of the questions for each respondent. Individual components are shown in the Supplementary file (Tables S1-S4).

Intentions to reduce work participation

We measured GPs intentions (in the next 5 years) to reduce work hours, leave medical work entirely, leave direct patient care, or to continue medical work but outside the UK, answered on on a 5-point scale with 1 indicating "none", 2 "slight", 3 "moderate", 4 "considerable" and 5 "high". We consider each of these components as separate outcomes.

Domains of working lives

The four domains of working lives were defined as follows:

Job satisfaction. Satisfaction with 10 different aspects of their job.[13] Ratings on a 7-point scale from 1 "extremely dissatisfied" and to 7 "extremely satisfied". The 10 answers were then averaged (minimum possible score of 1 and maximum possible score of 7).

Job stressors. Pressure experienceed from 13 job factors, rated on a 5-point scale; 1 "no pressure", 2 "slight pressure", 3 "moderate pressure", 4 "considerable pressure", 5 "high pressure". The 13 answers were averaged (minimum possible score of 1 and maximum possible score of 5).

Positive job attributes. Nine statements realting to 'positive' or desirable job aspects, where 1 indicates "strongly disagree", 2 "disagree", 3 "neutral", 4 "agree" and 5 "strongly agree". The nine answers were averaged (minimum possible score of 1 and maximum possible score of 5).

Negative job attributes. Four statements which relate to 'negative' or undesirable job aspects where 1 indicates "strongly disagree", 2 "disagree", 3 "neutral", 4 "agree" and 5 "strongly agree". The four answers were averaged (minimum possible score of 1 and maximum possible score of 5).

For peer review only - http://bmjopen.bmj.com/site/about/guidelines.xhtml

Covariates

We adjusted for: combinations of gender and age (five categories of 25-34, 35-44, 45-54, 55-64 and 65+ years); ethnicity (Black, Asian or minority ethnic group versus White ethnic group); practice urbanicity (proportion of patients living in an urban area,[16]and practice deprivation (proportion of patients living in an area in the most deprived quintile using an adjusted Index of Multiple Deprivation for England to ensure comparability with Scotland.[17]

Summary statistics and representativeness

The age, gender and partner status of respondents were compared to census data for GPs in England in December 2017[18] and in Scotland from the Information Services Division (ISD) Scotland in September 2018.[19] We also compared, separately for each country, the average proportions of patients in the most deprived quintile and in an urban area in the practices of the respondent GPs and all GPs. [16,19–22] The Scotland sample was more representative than the England sample, with women, younger doctors and non-partners being less under-represented in Scotland. GPs from more deprived and more urban practices were under-represented in the England sample (Supplementary file, Table S5). We adjust for these factors in the country comparisons.

Statistical analysis

Linear regression models with heteroscedasticity-robust standard errors to calculate point estimates and 95% confidence intervals for the differences of Scotland from England. In the adjusted models, age, gender, age and gender interaction, ethnicity, practice urbanicity and practice deprivation were included as covariates. We used average scores for the domains: job satisfaction, job stressors, positive job attributes and negative job attributes. The analysis was repeated on each separate question within these domains (see Supplementary file, Tables S1-S4). The questions were measured on ordered categorical scales and so we also checked the robustness of the adjusted

For peer review only - http://bmjopen.bmj.com/site/about/guidelines.xhtml

differences to the use of ordered probit regression models and results were qualitatively similar.

Results

Intentions to reduce work participation

GPs in Scotland reported lower likelihood of; reducing work hours (2.78 versus 3.54; adjusted difference -0.52; 95%CI -0.64 to -0.41), continuing medical work outside of the UK (1.41 versus 1.61; adjusted difference -0.2; 95%CI -0.28 to -0.12), leaving direct patient care (2.23 versus 2.93; adjusted difference -0.37; 95%CI -0.47 to -0.27), and leaving medical work entirely (2.11 versus 2.76; adjusted difference -0.32; 95%CI -0.42 to -0.22) compared with England (Table 1, Figure 1).

Domain scores

GPs in Scotland reported statistically significantly higher levels of satisfaction (5.27 versus 4.71; adjusted difference 0.50; 95%CI 0.42 to 0.59, lower levels of pressure (3.53 versus 3.86; adjusted difference -0.29; 95%CI -0.35 to -0.23), similar positive job attributes (3.19 versus 3.15; adjusted difference 0.01; 95%CI -0.04 to 0.06),) and less agreement in response to negative job attributes (4.03 versus 4.30; adjusted difference -0.27; 95%CI -0.31 to -0.22) (Table 2,

).

Scottish GPs had higher job satisfaction across all ten satisfaction domains, lower job stress for 12 of the 13 stressors (the exception being finding a locum), higher scores on six of the nine positive

For peer review only - http://bmjopen.bmj.com/site/about/guidelines.xhtml

job attributes (the exceptions being involvement in changes affecting their work, flexibility in working time and clear feedback about performance), and lower scores on all four of the negative job attributes (Figure 2). These differences remained in the adjusted regression analysis (see Supplementary file Tables S1-S4)

In both countries, almost every job stressor was (on average) rated in the range moderate to considerable. GPs in both countries disagreed or were neutral (on average) in their rating of five positive job attribute statements, with only slight average agreement in relation to having an interesting variety to work, knowing their responsibilities, having a choice in how to do the job, and being involved in changes introduced. On average, GPs in both countries agreed with all four negative job attribute statements (Figure 2) (see Supplementary file Tables S1-S4 for exact average values).

All differences between England and Scotland remained robust after controlling for GP characteristics, and practice urbanicity and deprivation. In the supplementary analyses, which restricted the sample in Scotland to the first 25.2% of returned surveys to match the total response rate in the English survey, there were only trivial differences in the results compared with those using the full sample (see Supplementary file Tables S6 and S7).

For peer review only - http://bmjopen.bmj.com/site/about/guidelines.xhtml

Discussion

Summary

This study has demonstrated that compared with GPs in England, GPs in Scotland have lower intentions to reduce work participation, as well as higher levels of job satisfaction, lower job stressors, and lower negative job attributes. These differences were of a reasonably large magnitude (one third to one half of a standard deviation), and thus likely to be meaningful in practice. It is possible that these differences relate, at least in part, to the recent changes in primary care in Scotland, including the new GP contract and the abolition (in April 2016) of the increasingly unpopular QOF [23].

However, notwithstanding these differences, GPs in both countries on average rated their work as moderately to considerably stressful. In both countries, the average response to statements relating to positive job attributes clustered around 'neither agree nor disagree', and the average response to statements relating to negative job attributes clustered around 'agree'. Although Scottish GPs had less intention to reduce work participation than English, significant minorities in both countries intended to reduce their hours or other work participation in the next five years.

Strengths and limitations

For peer review only - http://bmjopen.bmj.com/site/about/guidelines.xhtml

This is the first study to compare the working lives of GPs in Scotland and England using a set of common, validated questions on satisfaction, job characteristics and job pressures and using a similar data collection method. In both countries, largely nationally-representative samples were collected. We have adjusted the differences for respondents' age, gender, ethnicity and partner status, as well as the deprivation and urbanicity of the populations registered with the practices at which these GPs work.

The response rate was lower to the England survey. Evidence suggests that less satisfied GPs are more likely to respond to job satisfaction surveys [24]. If also true in this context, then the lower response rate in the English survey may mean it represents a pool of less satisfied English GPs and the differences could overestimate the true population differences. However, our supplementary analyses restricting the Scottish sample to the first 25.2% of returned surveys to match the total response rate in the English survey, showed only trivial differences compared to the full sample (see Supplementary file Tables S6 and S7).

Comparison with existing literature

The current 2017 survey in England work builds on previous English surveys on GP satisfaction using the same validated measures. This is the largest study ever on GPs' views on working lives in Scotland, and the first major study since the new Scottish GP contract was introduced in April 2018. Although Scotland generally had better scores than England, many of the absolute values in Scotland still give cause for concern. Almost a quarter (23.8%) of GPs in Scotland rated their chance of leaving direct patient care as 'considerable' or 'high'. This compares with a small survey conducted by RCGP Scotland in 2018 which found 26% of GPs felt they would be unlikely to be working in general practice in the next five years [25]. Thus, 'better' than England, does not necessarily mean 'good', and the findings give no room for complacency in either country.

A longitudinal study of GPs in the Wessex region of England conducted in 2014 and 2017 found worsening morale and higher intention to quit, with work intensity and amount being the most

For peer review only - http://bmjopen.bmj.com/site/about/guidelines.xhtml

common reasons given[26,27]. Such longitudinal surveys are required in Scotland, to understand GPs views as the new GP contract progresses, and to be able to compare with ongoing national surveys in England.

Implications for research and policy

We feel it is plausible to assume that (at least some of) the differences between GPs in Scotland and England in the present surveys were due to the timing and contract differences. Responsiveness of job satisfaction to new contracts has been shown before in the UK[28], although it is conceivable that differences between the two countries may have existed prior to the more recent policy changes; new Scottish contract reflects longer-standing differences from England in relation to NHS organisation, performance management and professional-managerial relationships [12].

In England, since the current survey was carried out, a new contract has been introduced in 2019 which focuses upon so-called 'Primary Care Networks' (PCNs) [29]. These are intended to cover between 30-50,000 people, although some are considerably larger[30]. The English contract also provides direct reimbursement for non-GP practitioners (e.g., pharmacists, physiotherapists, etc.) working within the PCN; payment for participating in a PCN; and access to incentive funding. Practices are required to deliver structured medication reviews, more support for Care Homes, early diagnosis of cancer, and anticipatory care for those most at risk of hospital admission. It is not yet clear what effect these changes will have on GPs' workload and job satisfaction.[31] The initial draft of the service specifications [32] was rejected by GPs as a significant burden of extra work [33,34].

Our study suggests that GPs in England feel additional pressures across all domains of practice compared to their Scottish colleagues. Whilst working together collaboratively as a PCN may increase satisfaction by engendering a sense of local community and support, it seems likely that overall satisfaction will only increase if perceived negative job attributes and job stressors also

For peer review only - http://bmjopen.bmj.com/site/about/guidelines.xhtml

reduce. It is therefore of vital importance to understand how the recruitment of non-GPs into general practice impacts upon different types of work.

The COVID-19 crisis has clearly had a significant impact on general practice in both countries, with practices switching to a 'triage first' model with markedly reduced face-to-face contact with patients.[35] Some of these changes may endure, but it is not at present clear which. It will be some time before we can understand the impact of COVID-19 on GP work, satisfaction and retention.

Further research is required to look at trends over time, as primary care reforms proceed across the UK in the coming years, and as the impact of COVID-19 evolves. In addition, research is required to understand how different elements of contract changes impact particular domains of satisfaction, and how workload changes in response to particular innovations.

For peer review only

Acknowledgements

We would like to thank all the GPs in England and Scotland who took part in this study.

Funding

The GP worklife survey in England is commissioned by the Department of Health and Social Care and carried out by the Policy Research Unit in Health and Social Care Systems and Commissioning (PRUComm). PRUComm is funded by the National Institute for Health Research (NIHR) Policy Research Programme (Ref: PR-PRU-1217-20801). The views expressed are those of the authors and not necessarily those of the NIHR, or the Department of Health

The GP worklife survey in Scotland was funded by internally by the Scottish School of Primary Care.

For peer review only - http://bmjopen.bmj.com/site/about/guidelines.xhtml

Author contributions

English survey: Matt Sutton and Kath Checkland devised the project. Helen Hayes, Matt Sutton and Jon Gibson were involved in the planning and design of methods. Jon Gibson, Kath Checkland and Matt Sutton were involved in the data collection for the English data. The data cleaning and analysis was carried out by Helen Hayes. Helen Hayes, Matt Sutton, Jon Gibson and Kath Checkland were involved in the writing of the manuscript.

Scottish survey: Stewart Mercer, Bruce Guthrie, John Gillies, and Bridie Fitzpatrick devised the project. Bruce Guthrie, Stewart Mercer, John Gillies, and Bridie Fitzpatrick were involved in the planning and design of the methods. Bridie Fitzpatrick led the data collection and data cleaning. Helen Hayes carried out the data analysis. Stewart Mercer led the revisions to the manuscript with input from Bruce Guthrie. Bridie Fitzpatrick and John Gillies read and revised the later versions.

All authors read and agreed the final version prior to submission. The corresponding author attests that all listed authors meet authorship criteria and that no others meeting the criteria have been omitted.

No competing interests

All authors have completed the ICMJE uniform disclosure form at www.icmje.org/coi_disclosure.pdf and declare: no support from any organisation for the submitted work; no financial relationships with any organisations that might have an interest in the submitted work in the previous three years; no other relationships or activities that could appear to have influenced the submitted work.

For peer review only

Patient and Public Involvement

There was no patient and public involvement in this study.

Data availability statement

No additional data available.

Dissemination declaration

We presented the findings of the study at the Scottish School of Primary Care conference on the 7th of May 2019.

Figure caption:

Figure 1: GPs Intentions to reduce work hours in Scotland and England

Figure 2: GPs mean responses to component questions of each domain in Scotland and England

Bibliography

1. Pathman DE, Konrad TR, Williams ES, Scheckler WE, Linzer M, Douglas J. Physician Job satisfaction, job dissatisfaction, and physician turnover. Vol. 51. 2002.

2. Scott A, Gravelle H, Simoens S, Bojke C, Sibbald B. Job satisfaction and quitting intentions: A structural model of British general practitioners. Br J Ind Relations. 2006 Sep;44(3):519–40.

3. Panagioti M, Geraghty K, Johnson J, Zhou A, Panagopoulou E, Chew-Graham C, et al. Association Between Physician Burnout and Patient Safety, Professionalism, and Patient Satisfaction. JAMA Intern Med [Internet]. 2018 Oct 1 [cited 2020 May 26];178(10):1317. Available from: https://jamanetwork.com/journals/jamainternalmedicine/fullarticle/2698144

For peer review only - http://bmjopen.bmj.com/site/about/guidelines.xhtml

4. West CP, Dyrbye LN, Shanafelt TD. Physician burnout: contributors, consequences and solutions. Vol. 283, Journal of Internal Medicine. Blackwell Publishing Ltd; 2018. p. 516–29.

5. Martin S, Davies E, Gershlick B. Under Pressure: What the Commonwealth Fund's 2015 International Survey of General Practitioners Means for the UK [Internet]. Analysis. 2016 [cited 2020 May 26]. Available from: http://www.health.org.uk/sites/health/files/UnderPressure.pdf

6. Fisher R, Turton C, Gershlick B, Alderwick H, Thorlby R. Feeling the strain: What The Commonwealth Fund's 2019 international survey of general practitioners means for the UK. The Health Foundation. 2020.

7. Roland M, Everington S. Tackling the crisis in general practice: If general practice fails, the whole NHS fails. Vol. 352, BMJ (Online). BMJ Publishing Group; 2016.

8. Hobbs FDR, Bankhead C, Mukhtar T, Stevens S, Perera-Salazar R, Holt T, et al. Clinical workload in UK primary care: a retrospective analysis of 100 million consultations in England, 2007–14. Lancet [Internet]. 2016 [cited 2020 May 26];387(10035):2323–30. Available from: https://www.sciencedirect.com/science/article/pii/S0140673616006206

9. Gibson J, Sutton M, Spooner S, Checkland K. Ninth National GP Worklife Survey. University of Manchester: Policy Research Unit in Commissioning and the Healthcare System Manchester Centre for Health Economics; 2018. p. 36 p.

10. NHS Digital. General Practice Workforce, Final 30 September 2018, Experimental statistics [Internet]. 2018 [cited 2020 May 26]. Available from: https://digital.nhs.uk/data-and-information/publications/statistical/general-practice-workforce-archive/final-30-september-2018-experimental-statistics

11. Smith GI, Mercer SW, Gillies JCM, McDevitt A. Improving together: A new quality framework for GP clusters in Scotland. Vol. 67, British Journal of General Practice. Royal College of General Practitioners; 2017. p. 294–5.

12. The Scottish Government and BMA. The 2018 General Medical Services Contract in Scotland [Internet]. 2017 [cited 2020 May 26]. Available from: https://www.gov.scot/publications/gms-

For peer review only

contract-scotland/

13. Warr P, Cook J, Wall T. Scales for the measurement of some work attitudes and aspects of psychological well-being. J Occup Psychol. 1979 Jun 1;52(2):129–48.

14. Sibbald B, Bojke C, Gravelle H. National survey of job satisfaction and retirement intentions among general practitioners in England. Br Med J. 2003 Jan 4;326(7379):22–4.

15. Mercer S. GP Survey - Scottish Findings. 2019.

16. ONS. 2011 rural/urban classification [Internet]. 2016 [cited 2020 May 26]. Available from: https://www.ons.gov.uk/methodology/geography/geographicalproducts/ruralurbanclassifications/2011ruralurbanclassification

17. Abel GA, Barclay ME, Payne RA. Adjusted indices of multiple deprivation to enable comparisons within and between constituent countries of the UK including an illustration using mortality rates. BMJ Open [Internet]. 2016 [cited 2020 May 26];6(11). Available from: http://bmjopen.bmj.com/

18. NHS Digital. General and Personal Medical Services, England As at 31 December 2017, Provisional Experimental statistics [Internet]. 2018 [cited 2020 May 26]. Available from: https://digital.nhs.uk/data-and-information/publications/statistical/general-practice-workforce-archive/as-at-31-december-2017-provisional-experimental-statistics

19. ISD Scotland. General Practice - GP Workforce and practice list sizes 2008-2018 [Internet]. 2018 [cited 2020 May 26]. Available from: https://www.isdscotland.org/Health-Topics/General-Practice/Publications/2018-12-11/2018-12-11-GPWorkforce2018-Report.pdf

20. ISD Scotland. List of GPs in post and their practice contact details [Internet]. 2019. Available from: https://www.isdscotland.org/Health-Topics/General-Practice/Workforce-and-Practice-Populations/

21. Abel G, Payne R, Barclay M. UK Deprivation Indices. University of Bristol; 2016.

22. NHS Digital. Patients Registered at a GP Practice, January 2018; Special Topic - Patients age group comparison [Internet]. 2018 [cited 2020 May 26]. Available from:

For peer review only - http://bmjopen.bmj.com/site/about/guidelines.xhtml

https://digital.nhs.uk/data-and-information/publications/statistical/patients-registered-at-a-gp-practice/january-2018

23. Roland M, Guthrie B. Quality and Outcomes Framework: What have we learnt? BMJ. 2016 Aug 4;354.

24. Gravelle H, Hole AR, Hossain MI. Response bias in job satisfaction surveys: English general practitioners. Discussion papers in economics, no. 2008/24; 2008.

25. RCGP Scotland. From the Frontline - The changing landscape of Scottish general practice. 2019.

26. Owen K, Hopkins T, Shortland T, Dale J. GP retention in the UK: A worsening crisis. Findings from a cross-sectional survey. BMJ Open. 2019 Feb 1;9(2):e026048.

27. Dale J, Potter R, Owen K, Parsons N, Realpe A, Leach J. Retaining the general practitioner workforce in England: What matters to GPs? A cross-sectional study. BMC Fam Pract [Internet]. 2015 Oct 16 [cited 2020 Sep 21];16(1):140. Available from: http://www.biomedcentral.com/1471-2296/16/140

28. Whalley D, Gravelle H, Sibbald B. Effect of the new contract on GPs' working lives and perceptions of quality of care: A longitudinal survey. Br J Gen Pract. 2008 Jan 1;58(546):8–14.

29. NHS England, BMA. Investment and evolution: A five-year framework for GP contract reform to implement The NHS Long Term Plan. 2019;

30. Morciano M, Checkland K, Hammond J, Lau Y-S, Sutton M. Variability in size and characteristics of England's Primary Care Networks: observational study [Internet]. British Journal of General Practice; 2020 [cited 2020 May 29]. Available from: https://www.research.manchester.ac.uk/portal/en/publications/variability-in-size-and-characteristics-of-englands-primary-care-networks-observational-study(4f9596ac-3fdd-4f05-82a2-b61893733601).html

31. Sibbald B, Shen J, McBride A. Changing the skill-mix of the health care workforce. J Health Serv Res Policy [Internet]. 2004 Jan [cited 2020 Jan 19];9 Suppl 1:28–38. Available from:

For peer review only

For peer review only - http://bmjopen.bmj.com/site/about/guidelines.xhtml

http://www.ncbi.nlm.nih.gov/pubmed/15006226

32. NHS England and NHS Improvement. Network Contract Direct Enhanced Service - Draft Service Specifications. 2019.

33. Murray R. GPs condemn new specifications for primary care networks. Vol. 368, The BMJ. BMJ Publishing Group; 2020.

34. Wilson T, Lewis R. Primary care networks: Well intentioned but overambitious. Vol. 366, The BMJ. BMJ Publishing Group; 2019.

35. NHS England, NHS Improvement. Guidance and standard operating procedures: General practice in the context of coronavirus (COVID-19). 2020.

For peer review only

Table 1: Differences between Scotland and England in intentions to reduce work participation

	Scotland (Mean, SD)	England (Mean, SD)	Unadjusted difference (Coef., 95% CI)	Adjusted difference (Coef., 95% CI)

For peer review only - http://bmjopen.bmj.com/site/about/guidelines.xhtml

Reduce your work hours within five years?	2.78 (1.56)	3.54 (1.49)	-0.76 [-0.88 to -0.64]	-0.52 [-0.64 to -0.41]
Leave medical work entirely within five years?	2.11 (1.49)	2.76 (1.63)	-0.65 [-0.78 to -0.52]	-0.32 [-0.42 to -0.22]
Leave direct patient care within five years?	2.23 (1.51)	2.93 (1.59)	-0.70 [-0.82 to -0.58]	-0.37 [-0.47 to -0.27]
Continue with medical work but outside the UK within five years?	1.41 (0.84)	1.61 (1.03)	-0.21 [-0.28 to -0.13]	-0.20 [-0.28 to -0.12]

Notes: N=2,927. All four measures of intentions to reduce work participation are measured on a five-point scale, 1="none" to 5="high". Columns 4 and 5 are coefficients from linear regressions with heteroscedasticity-robust standard errors. Adjusted differences control for gender and age category interactions, ethnicity, partner status, and practice deprivation and urbanicity.

For peer review only

Table 2: Differences between Scotland and England in satisfaction, job stressors and job attributes

	Scotland (Mean, SD)	England (Mean, SD)	Unadjusted difference (Coef., 95% CI)	Adjusted difference (Coef., 95% CI)
Satisfaction	5.27 (0.97)	4.71 (1.07)	0.55 [0.47 to 0.63]	0.50 [0.42 to 0.59]
Job stressors	3.53 (0.75)	3.86 (0.66)	-0.34 [-0.39 to -0.28]	-0.29 [-0.35 to -0.23]
Positive job attributes	3.19 (0.57)	3.15 (0.62)	0.037 [-0.011 to 0.085]	0.013 [-0.038 to 0.063]

For peer review only - http://bmjopen.bmj.com/site/about/guidelines.xhtml

Negative job attributes	4.03 (0.64)	4.30 (0.57)	-0.27 [-0.32 to -0.23]	-0.27 [-0.31 to -0.22]

Notes: N=2,927. Satisfaction is measured 1 (extremely dissatisfied) to 7 (extremely satisfied). Job stressors are measured 1 (no pressure) to 5 (high pressure). Job attributes are measured 1 (strongly disagree) to 5 (strongly agree) with 3 neutral. Columns 4 and 5 are coefficients from linear regressions with robust standard errors. Adjusted differences control for age, gender, age x gender interaction, ethnicity, partner, and practice deprivation and urbanicity.

For peer review only

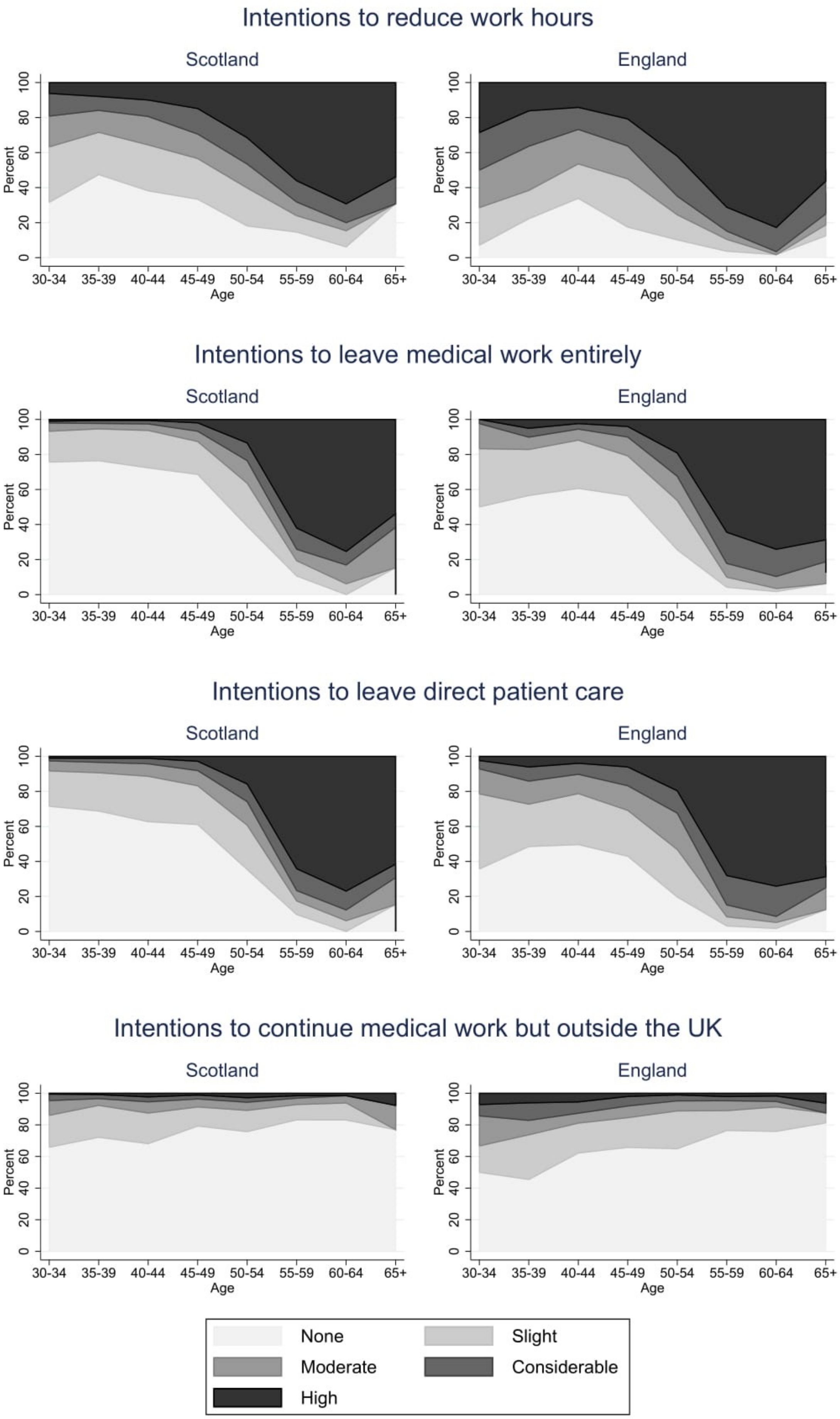
Intentions to reduce work hours
Scotland
England
Intentions to leave medical work entirely
Scotland
England
Intentions to leave direct patient care
Scotland
England
Intentions to continue medical work but outside the UK
Scotland
England
Percent
0
20
40
60
80
100
Age
30-34
35-39
40-44
45-49
50-54
55-59
60-64
65+
None
Slight
Moderate
Considerable
High

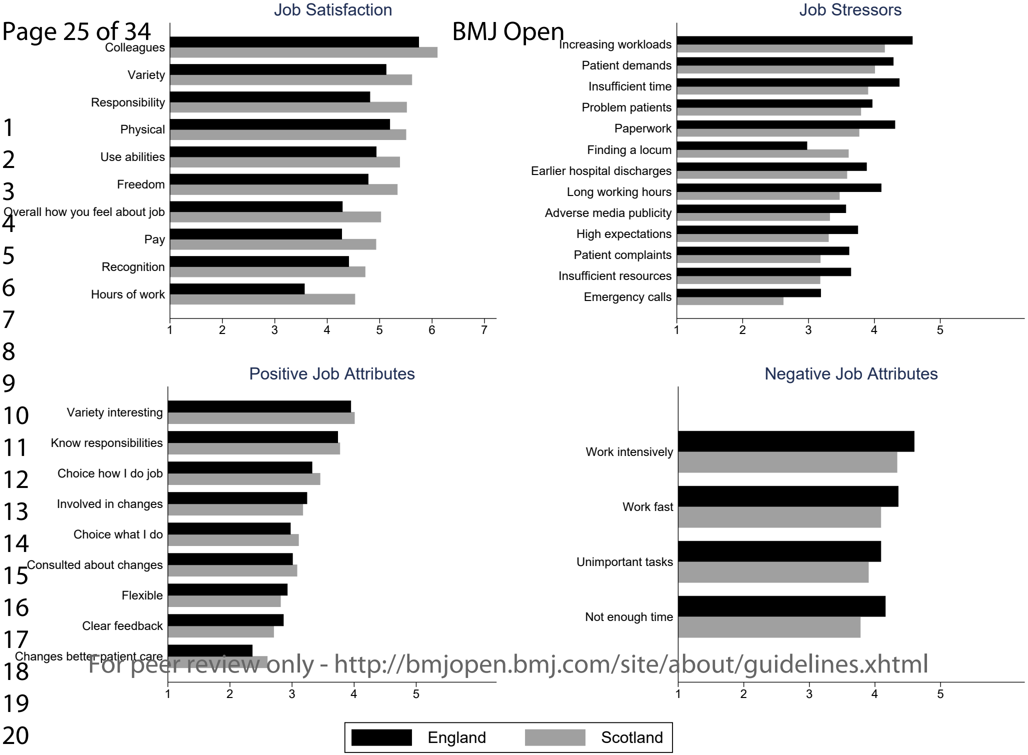

For peer review only - http://bmjopen.bmj.com/site/about/guidelines.xhtml

SUPPLEMENTARY FILE

For peer review only

Table S1: Country differences in each domain of job satisfaction

Job satisfaction domain	Scotland (Mean, SD)	England (Mean, SD)	Unadjusted difference (Coeff, CI)	Adjusted difference (Coeff, CI)
Your colleagues and fellow workers	6.10 (1.14)	5.75 (1.23)	0.35 [0.26 to 0.45]	0.30 [0.20 to 0.39]
Amount of variety in your job	5.61 (1.20)	5.12 (1.38)	0.49 [0.39 to 0.60]	0.44 [0.34 to 0.55]
Amount of responsibility you are given	5.51 (1.43)	4.81 (1.68)	0.70 [0.57 to 0.83]	0.61 [0.48 to 0.74]
Physical working conditions	5.50 (1.45)	5.19 (1.45)	0.31 [0.19 to 0.42]	0.29 [0.18 to 0.41]
Opportunities to use your abilities	5.38 (1.26)	4.93 (1.45)	0.45 [0.34 to 0.56]	0.40 [0.29 to 0.52]
Freedom to choose your own method of working	5.34 (1.27)	4.78 (1.45)	0.56 [0.45 to 0.67]	0.53 [0.41 to 0.64]
Taking everything into consideration, how do you feel about your job	5.02 (1.34)	4.29 (1.49)	0.73 [0.62 to 0.85]	0.66 [0.54 to 0.78]
Your remuneration	4.93 (1.57)	4.28 (1.65)	0.65 [0.53 to 0.78]	0.60 [0.47 to 0.74]
Recognition you get for good work	4.72 (1.54)	4.41 (1.58)	0.31 [0.19 to 0.44]	0.28 [0.15 to 0.41]
Your hours of work	4.53 (1.66)	3.57 (1.79)	0.96 [0.82 to 1.10]	0.88 [0.74 to 1.03]

Notes: N=2,927. Satisfaction components are measured on a seven-point scale. Columns 4 and 5 are coefficients from linear regressions with robust standard errors. Adjusted differences control for age, gender, age x gender interaction, ethnicity, partner, and practice deprivation and urbanicity.

For peer review only

Table S2: Country differences in each job stressor

Job stressor	Scotland (Mean, SD)	England (Mean, SD)	Unadjusted difference (Coeff, CI)	Adjusted difference (Coeff, CI)
Increasing workloads	4.15 (0.93)	4.58 (0.68)	-0.42 [-0.48 to -0.36]	-0.41 [-0.47 to -0.35]
Increased demands from patients	4.01 (0.91)	4.29 (0.73)	-0.28 [-0.34 to -0.22]	-0.27 [-0.34 to -0.21]
Having insufficient time to do justice to the job	3.90 (1.04)	4.38 (0.83)	-0.48 [-0.55 to -0.41]	-0.47 [-0.54 to -0.39]
Dealing with problem patients	3.79 (0.97)	3.97 (0.88)	-0.17 [-0.24 to -0.10]	-0.16 [-0.24 to -0.084]
Paperwork	3.77 (0.97)	4.31 (0.87)	-0.54 [-0.62 to -0.47]	-0.52 [-0.59 to -0.44]
Finding a locum	3.61 (1.42)	2.98 (1.41)	0.63 [0.52 to 0.74]	0.75 [0.64 to 0.86]
Dealing with earlier discharges from hospital	3.58 (0.99)	3.88 (0.93)	-0.30 [-0.37 to -0.22]	-0.24 [-0.32 to -0.16]
Long working hours	3.47 (1.16)	4.10 (1.04)	-0.63 [-0.72 to -0.55]	-0.59 [-0.68 to -0.50]
Adverse publicity by the media	3.32 (1.25)	3.57 (1.22)	-0.25 [-0.34 to -0.15]	-0.21 [-0.31 to -0.11]
Unrealistically high expectation of role by others	3.30 (1.20)	3.75 (1.12)	-0.45 [-0.54 to -0.36]	-0.38 [-0.48 to -0.29]
Worrying about patient complaints/litigation	3.18 (1.13)	3.61 (1.12)	-0.44 [-0.52 to -0.35]	-0.38 [-0.48 to -0.29]
Insufficient resources within the practice	3.17 (1.16)	3.64 (1.15)	-0.47 [-0.56 to -0.38]	-0.38 [-0.47 to -0.28]
Interruptions by emergency calls during surgery	2.62 (1.10)	3.18 (1.11)	-0.57 [-0.66 to -0.48]	-0.52 [-0.61 to -0.43]

Notes: N=2,927. Stressors components are measured on a five-point scale. Columns 4 and 5 are coefficients from linear regressions with robust standard errors. Adjusted differences control for age, gender, age x gender interaction, ethnicity, partner, and practice deprivation and urbanicity.

For peer review only

Table S3: Country differences in each positive job attribute

Positive job attribute	Scotland (Mean, SD)	England (Mean, SD)	Unadjusted difference (Coeff, CI)	Adjusted difference (Coeff, CI)
My job provides me with a variety of interesting things	4.01 (0.73)	3.95 (0.78)	0.059 [-0.0019 to 0.12]	0.032 [-0.029 to 0.093]
I always know what my work responsibilities are	3.77 (0.85)	3.74 (0.93)	0.034 [-0.037 to 0.11]	0.0022 [-0.073 to 0.077]
I have a choice in deciding how I do my job	3.45 (0.94)	3.32 (1.00)	0.13 [0.051 to 0.21]	0.091 [0.011 to 0.17]
I am involved in deciding on the changes introduced that affect my work	3.17 (1.10)	3.24 (1.22)	-0.067 [-0.16 to 0.027]	-0.088 [-0.18 to 0.0088]
I have a choice in deciding what I do at work	3.10 (0.99)	2.97 (1.01)	0.13 [0.052 to 0.21]	0.10 [0.021 to 0.19]
I am consulted about changes that affect my work	3.08 (1.10)	3.01 (1.28)	0.072 [-0.025 to 0.17]	0.0052 [-0.096 to 0.11]
My working time can be flexible	2.81 (1.06)	2.92 (1.05)	-0.11 [-0.19 to -0.026]	-0.12 [-0.21 to -0.037]
I get clear feedback about how well I am doing my job	2.70 (0.92)	2.86 (0.96)	-0.16 [-0.23 to -0.082]	-0.13 [-0.21 to -0.049]
Changes to my job in the last year have led to better patient care	2.60 (0.95)	2.36 (0.97)	0.24 [0.17 to 0.32]	0.22 [0.14 to 0.30]

Notes: N=2,927. Positive job attributes components are measured on a five-point scale. Columns 4 and 5 are coefficients from linear regressions with robust standard errors. Adjusted differences control for age, gender, age x gender interaction, ethnicity, partner, and practice deprivation and urbanicity.

For peer review only

Table S4: Country differences in each negative job attribute

Negative job attributes domain	Scotland (Mean, SD)	England (Mean, SD)	Unadjusted difference (Coeff, CI)	Adjusted difference (Coeff, CI)
I have to work very intensively	4.34 (0.73)	4.60 (0.62)	-0.26 [-0.31 to -0.21]	-0.26 [-0.32 to -0.21]
I have to work very fast	4.09 (0.83)	4.35 (0.76)	-0.26 [-0.33 to -0.20]	-0.26 [-0.33 to -0.20]
I am required to do unimportant tasks which prevent me completing more important ones	3.90 (0.97)	4.09 (0.97)	-0.19 [-0.27 to -0.11]	-0.18 [-0.26 to -0.10]
I do not have time to carry out all my work	3.78 (1.02)	4.16 (0.98)	-0.38 [-0.46 to -0.30]	-0.36 [-0.44 to -0.28]

Notes: N=2,927. Negative job attributes components are measured on a five-point scale. Columns 4 and 5 are coefficients from linear regressions with robust standard errors. Adjusted differences control for age, gender, age x gender interaction, ethnicity, partner, and practice deprivation and urbanicity.

For peer review only

Table S5: Summary statistics for the covariates showing representativeness of the two surveys

	Scotland		England	
Variable names	Survey GPs (n=2,048) %	All GPs (Sep 2018) (n=4,366) %	Survey GPs (n=879) %	All GPs (Dec 2017) (n=34,549 %
Female	58%	58%	51%	54%
GP Partner	84%	78%	87%	66%
Black, Asian and Minority Ethnicity	9%	n/a	20%	n/a
Age category:				
<35 years	10%	13%	5%	12%
35-44 years	34%	34%	26%	34%
45-54 years	36%	33%	38%	31%
55-64 years	19%	18%	28%	19%
65+ years	1%	1%	3%	4%
Proportion of patients in an urban area	65%	66%	77%	81%
Proportion of patients in most deprived quintile	20%	19%	16%	19%

Notes: All data are presented for salaried and partnered GPs only. This is with the exception of population figures for deprivation and urbanicity indicators for Scotland, as GP headcount data by practice used to create this figure is only available for all GPs (including registrars, retainers and locums).
For deprivation, we use data on the number of patients living in each deprivation quintile registered with each practice. For Scotland, number of patients in each deprivation quintile is publicly available. For England, this is created using data on LSOA level quintiles of IMD and the number of patients in each LSOA.
For urbanicity, we use the distribution of patients in urban and rural areas. For Scotland, number of patients in each practice in each urban/rural classification is publicly available. For England this was created using the 2011 urban/rural classification by LSOA and the number of patients in each practice in each LSOA.
Ethnicity data is unavailable at the population level.

For peer review only

Table S6: Restricted sample analysis of differences between Scotland and England in intentions to reduce work participation

	Scotland (Mean, SD)	England (Mean, SD)	Unadjusted difference (Coef., 95% CI)	Adjusted difference (Coef., 95% CI)
Reduce your work hours within five years?	2.84 (1.58)	3.54 (1.49)	-0.70 [-0.84 to -0.55]	-0.53 [-0.66 to -0.39]
Leave medical work entirely within five years?	2.20 (1.53)	2.76 (1.63)	-0.55 [-0.70 to -0.41]	-0.31 [-0.42 to -0.20]
Leave direct patient care within five years?	2.33 (1.54)	2.93 (1.59)	-0.60 [-0.74 to -0.45]	-0.36 [-0.47 to -0.24]
Continue with medical work but outside the UK within five years?	1.37 (0.79)	1.61 (1.03)	-0.25 [-0.33 to -0.16]	-0.22 [-0.30 to -0.13]

Notes: N=1,800. Intentions to reduce work participation indicators are measured on a five-point scale. Columns 4 and 5 are coefficients from linear regressions with robust standard errors. Adjusted differences control for age, gender, age x gender interaction, ethnicity, partner, and practice deprivation and urbanicity.
Analysis is based on the first 25.2% of respondents in the Scottish sample to match the returned English sample.

For peer review only

Table S7: Restricted sample analysis of differences between Scotland and England across all satisfaction, job stressors, and job attributes domains

	Scotland (Mean, SD)	England (Mean, SD)	Unadjusted difference (Coef., 95% CI)	Adjusted difference (Coef., 95% CI)
Satisfaction	5.29 (0.98)	4.71 (1.07)	0.58 [0.49 to 0.68]	0.51 [0.41 to 0.61]
Job stressors	3.53 (0.78)	3.86 (0.66)	-0.33 [-0.40 to -0.26]	-0.28 [-0.35 to -0.21]
Positive job attributes	3.19 (0.60)	3.15 (0.62)	0.037 [-0.020 to 0.093]	0.0054 [-0.053 to 0.064]
Negative job attributes	4.01 (0.66)	4.30 (0.57)	-0.29 [-0.35 to -0.23]	-0.28 [-0.34 to -0.22]

Notes: N=1,800. Satisfaction is measured 1-7. Job stressors and job attributes are measured 1-5. Columns 4 and 5 are coefficients from linear regressions with robust standard errors. Adjusted differences control for age, gender, age x gender interaction, ethnicity, partner, and practice deprivation and urbanicity.
Analysis is based on the first 25.2% of respondents in the Scottish sample to match the returned English sample.

For peer review only

STROBE Statement—Checklist of items that should be included in reports of ***cross-sectional studies***

	Item No	Recommendation	Page No
**Title and abstract**	1	(*a*) Indicate the study's design with a commonly used term in the title or the abstract	1
		(*b*) Provide in the abstract an informative and balanced summary of what was done and what was found	2,3
**Introduction**			
Background/rationale	2	Explain the scientific background and rationale for the investigation being reported	4,5
Objectives	3	State specific objectives, including any prespecified hypotheses	5
**Methods**			
Study design	4	Present key elements of study design early in the paper	7
Setting	5	Describe the setting, locations, and relevant dates, including periods of recruitment, exposure, follow-up, and data collection	7
Participants	6	(*a*) Give the eligibility criteria, and the sources and methods of selection of participants	7
Variables	7	Clearly define all outcomes, exposures, predictors, potential confounders, and effect modifiers. Give diagnostic criteria, if applicable	7,8
Data sources/ measurement	8*	For each variable of interest, give sources of data and details of methods of assessment (measurement). Describe comparability of assessment methods if there is more than one group	7,8
Bias	9	Describe any efforts to address potential sources of bias	9
Study size	10	Explain how the study size was arrived at	6,7
Quantitative variables	11	Explain how quantitative variables were handled in the analyses. If applicable, describe which groupings were chosen and why	9
Statistical methods	12	(*a*) Describe all statistical methods, including those used to control for confounding	9
		(*b*) Describe any methods used to examine subgroups and interactions	9
		(*c*) Explain how missing data were addressed	9
		(*d*) If applicable, describe analytical methods taking account of sampling strategy	9
		(*e*) Describe any sensitivity analyses	
**Results**			
Participants	13*	(a) Report numbers of individuals at each stage of study—eg numbers potentially eligible, examined for eligibility, confirmed eligible, included in the study, completing follow-up, and analysed	Suppl. File
		(b) Give reasons for non-participation at each stage	N/A
		(c) Consider use of a flow diagram	N/A
Descriptive data	14*	(a) Give characteristics of study participants (eg demographic, clinical, social) and information on exposures and potential confounders	Suppl. File
		(b) Indicate number of participants with missing data for each variable of interest	7
Outcome data	15*	Report numbers of outcome events or summary measures	10
Main results	16	(*a*) Give unadjusted estimates and, if applicable, confounder-adjusted estimates and their precision (eg, 95% confidence interval). Make clear which confounders were adjusted for and why they were included	10,11 +

For peer review only - http://bmjopen.bmj.com/site/about/guidelines.xhtml

			Suppl. File
		(*b*) Report category boundaries when continuous variables were categorized	10,11
		(*c*) If relevant, consider translating estimates of relative risk into absolute risk for a meaningful time period	N/A
Other analyses	17	Report other analyses done—eg analyses of subgroups and interactions, and sensitivity analyses	10,11
**Discussion**			
Key results	18	Summarise key results with reference to study objectives	12
Limitations	19	Discuss limitations of the study, taking into account sources of potential bias or imprecision. Discuss both direction and magnitude of any potential bias	12,13
Interpretation	20	Give a cautious overall interpretation of results considering objectives, limitations, multiplicity of analyses, results from similar studies, and other relevant evidence	13-15
Generalisability	21	Discuss the generalisability (external validity) of the study results	14,15
**Other information**			
Funding	22	Give the source of funding and the role of the funders for the present study and, if applicable, for the original study on which the present article is based	16

*Give information separately for exposed and unexposed groups.

Note: An Explanation and Elaboration article discusses each checklist item and gives methodological background and published examples of transparent reporting. The STROBE checklist is best used in conjunction with this article (freely available on the Web sites of PLoS Medicine at http://www.plosmedicine.org/, Annals of Internal Medicine at http://www.annals.org/, and Epidemiology at http://www.epidem.com/). Information on the STROBE Initiative is available at www.strobe-statement.org.

For peer review only
